# A Programmable Eukaryotic Argonaute Nuclease with Dual DNA and RNA Cleavage Activity from Thermophilic Fungus *Thermochaetoides thermophila*

**DOI:** 10.1002/smsc.202500237

**Published:** 2026-03-15

**Authors:** Zhizhao Chen, Fei Wang, Xiaolan Yu, Yang Liu, Lixin Ma

**Affiliations:** ^1^ State Key Laboratory of Biocatalysis and Enzyme Engineering Hubei Key Laboratory of Industrial Biotechnology School of Life Sciences Hubei University Wuhan Hubei China

**Keywords:** DNA cleavage, eukaryotic argonaute, RNA cleavage

## Abstract

Eukaryotic Argonautes (eAgos) are well‐known for their role in utilizing RNA guides to recognize and cleave RNA targets, thereby participating in posttranscriptional regulation and RNA interference. However, it remains unclear whether certain eAgos can also cleave DNA at mesophilic temperatures. Here, we describe a unique eAgo nuclease, TtpAgo, from the thermophilic fungus *Thermochaetoides thermophila* DSM 1495 that can be programmed with either DNA or RNA guides and can cleave both DNA and RNA targets at mesophilic temperatures in vitro. TtpAgo binds 12–30 nt long 5′phosphorylated or 5′hydroxylated guide molecules without strict sequence specificity and functions across a wide temperature range. Mismatches between guide and target sequences greatly impact cleavage efficiency and precision, influenced by mismatch position and nucleic acid characteristics. TtpAgo performs programmable cleavage of highly structured RNA at 37°C and 55°C. These properties of TtpAgo redefine the catalytic scope of eAgos and provide a versatile tool for manipulating nucleic acids in vitro.

## Graphical abstract

This work demonstrates a unique eukaryotic Argonaute nuclease, TtpAgo, from *Thermochaetoides thermophila* DSM 1495 that can be programmed with either DNA or RNA guides and can cleave both DNA and RNA targets at mesophilic temperatures in vitro. TtpAgo binds 12–30 nt long 5^′^P or 5^′^OH guide molecules without strict sequence specificity, thus provides a versatile tool for manipulating nucleic acids.

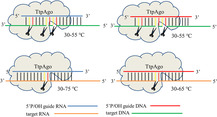

## 1 Introduction

Argonaute (Ago) proteins are evolutionarily conserved nucleic acid‐guided endonucleases central to gene silencing pathways across all domains of life [1, 2, 3, 4]. In eukaryotes, Ago family members, such as human Ago2, are well‐characterized components of the RNA‐induced silencing complex, where they bind small RNAs to guide sequence‐specific cleavage of complementary RNA targets [5, 6, 7]. This canonical RNA‐targeting activity underpins RNA interference (RNAi), a critical mechanism for posttranscriptional regulation and defense against viral invaders [8, 9, 10]. By contrast, prokaryotic Agos (pAgos) exhibit remarkable mechanistic diversity. While the well‐characterized long‐A pAgos (e.g., *Thermus thermophilus* Ago) always employ DNA guides to cleave DNA targets and defend against invasive genetic elements [11, 12, 13], this represents only one operational mode. Our research and that of others have identified various long‐A pAgos with distinct catalytic activities, such as KmAgo (*Kurthia massiliensis*) [14, 15] and MhAgo (*Marinitoga hydrogenitolerans*) [16] capable of DNA‐guided DNA cleavage, MbpAgo (*Mucilaginibacter paludism*) [17] and VbAgo (*Verrucomicrobia*) [18] showing a preference for DNA‐guided RNA cleavage, along with MpAgo (*Marinotoga piezophile*) [19] having a preference for RNA‐guided DNA cleavage. Moreover, recent studies have uncovered additional variants with distinct functionalities, including those mediating RNA‐guided RNA targeting [20]. Meanwhile, the majority of pAgos actually belong to long‐B and short subtypes that predominantly use RNA guides to target‐but not cleave‐DNA [21, 22]. For decades, this functional dichotomy—eukaryotic Agos (eAgos) targeting RNA [6, 7, 23, 24, 25, 26], prokaryotic counterparts targeting DNA and RNA [15, 27, 28, 29, 30]—has framed our understanding of Ago biology, leaving unresolved whether eukaryotic systems evolved analogous DNA‐targeting capabilities [31, 32].

Earlier studies have hinted at unappreciated diversity in eAgo functions [33]. Some noncanonical roles, such as DNA binding in transcriptional silencing or interactions with chromatin [34, 35, 36, 37], have been reported. Recently, our group reported eAgo from *Chaetomium* sp. MPI‐CAGE‐AT‐0009 (CsAgo) can utilize gDNAs to cleave DNA at high temperatures [38], but catalytic DNA cleavage by an eAgo at mesophilic temperatures in vitro remains undocumented [31]. This gap persists despite structural similarities between pAgos and eukaryotic homologs, suggesting potential latent functionalities. For instance, certain eAgo subtypes may retain evolutionarily dormant DNA‐processing activities under specific cellular conditions. Furthermore, the biotechnological utility of pAgos as programmable DNA‐guided DNA cutters has spurred interest in discovering analogous systems in eukaryotes [39, 40, 41], which could offer advantages such as enhanced compatibility with endogenous cellular machinery. Whether such enzymes exist—and how their mechanisms compare to established RNA‐targeting eAgos—remains a pivotal unanswered question.

Here, we report the discovery of an eAgo, derived from the thermophilic fungus *Thermochaetoides thermophila* DSM 1495 (TtpAgo), which exhibits the ability to selectively cleave DNA and RNA targets in a programmable and sequence‐specific manner in vitro. In vitro reconstitution assays demonstrate that this Ago utilizes small DNA guides and RNA guides to cleave complementary DNA substrates. Strikingly, TtpAgo retains robust RNA cleavage activity, a feature absence in most DNA‐targeting pAgos. This dual activity challenges the long‐standing functional divide between prokaryotic and eukaryotic Ago systems.

## 2 Results

### 2.1 TtpAgo Can use All Four Types of Guides to Target Both ssDNA and RNA In Vitro

Currently, all characterized eAgos are derived from organisms that thrive at physiological temperatures [6, 26, 42]. We selected the protein sequence of QDE2 (AAF43641.1; an eAgo from *Neurospora crassa*) as a query and used the BLASTp web interface to search for thermophilic eAgos. From the database, we identified TtpAgo (XP_006694702.1; *Thermochaetoides thermophila* DSM 1495), which shares 46.71% sequence identity with QDE2. We used the maximum likelihood method to perform a phylogenetic tree comparison of TtpAgo with the already characterized Ago. The results showed that TtpAgo was closely related to the fungus Ago, and relatively distantly related to higher animals and plants and the PIWI family (Figure S1A). Bioinformatic analysis revealed that TtpAgo contains the canonical catalytic tetrad in the PIWI domain (residues D765, E809, D843, and D988; see Figure S1G and Figure S8B). Similarly, we analyzed the conserved amino acids of the MID domain and PAZ domain and found that Y696, K700, and K739 are as conserved as other Agos in the MID structure, but V712 is different from other Agos (Figure S8A). However, the residues corresponding to positions 430–431 in the PAZ domain, which are conserved as RK in other eAgos, are DE in TtpAgo, representing an opposite charge. The impact of this difference on TtpAgo's activity remains unclear (Figure S8C). TtpAgo and TtpAgo‐QM (Quadruple mutant; D765A/E809A/D843A/D988A) were expressed and purified to investigate biochemical properties (Figure S1B,C).

Following initial Ni‐NTA purification, TtpAgo preparations contained both high‐molecular weight DNA and small RNAs (Figure S1E). However, upon further purification by heparin affinity chromatography and size‐exclusion chromatography, the associated nucleic acid profile changed markedly: the DNA was largely eliminated, whereas small RNAs remained detectable (Figure S1D). Given the known tendency of Ni‐NTA resins to nonspecifically retain negatively charged nucleic acids, the presence of DNA after Ni‐NTA purification is likely due to nonspecific binding to the resin rather than a specific association with TtpAgo. Consistent with this interpretation, similar DNA copurification was observed when superfolder GFP (sfGFP) was purified using the same protocol (Figure S1F). In contrast, the persistence of small RNAs after additional purification steps suggests a more stable association with TtpAgo, consistent with previous reports on RNA binding by eAgos [42, 43]. RNA sequencing of the copurified small RNAs revealed an enrichment of transcripts derived from the lacI gene (Table S2), which is likely influenced by the expression system used in this study.

**FIGURE 1:**
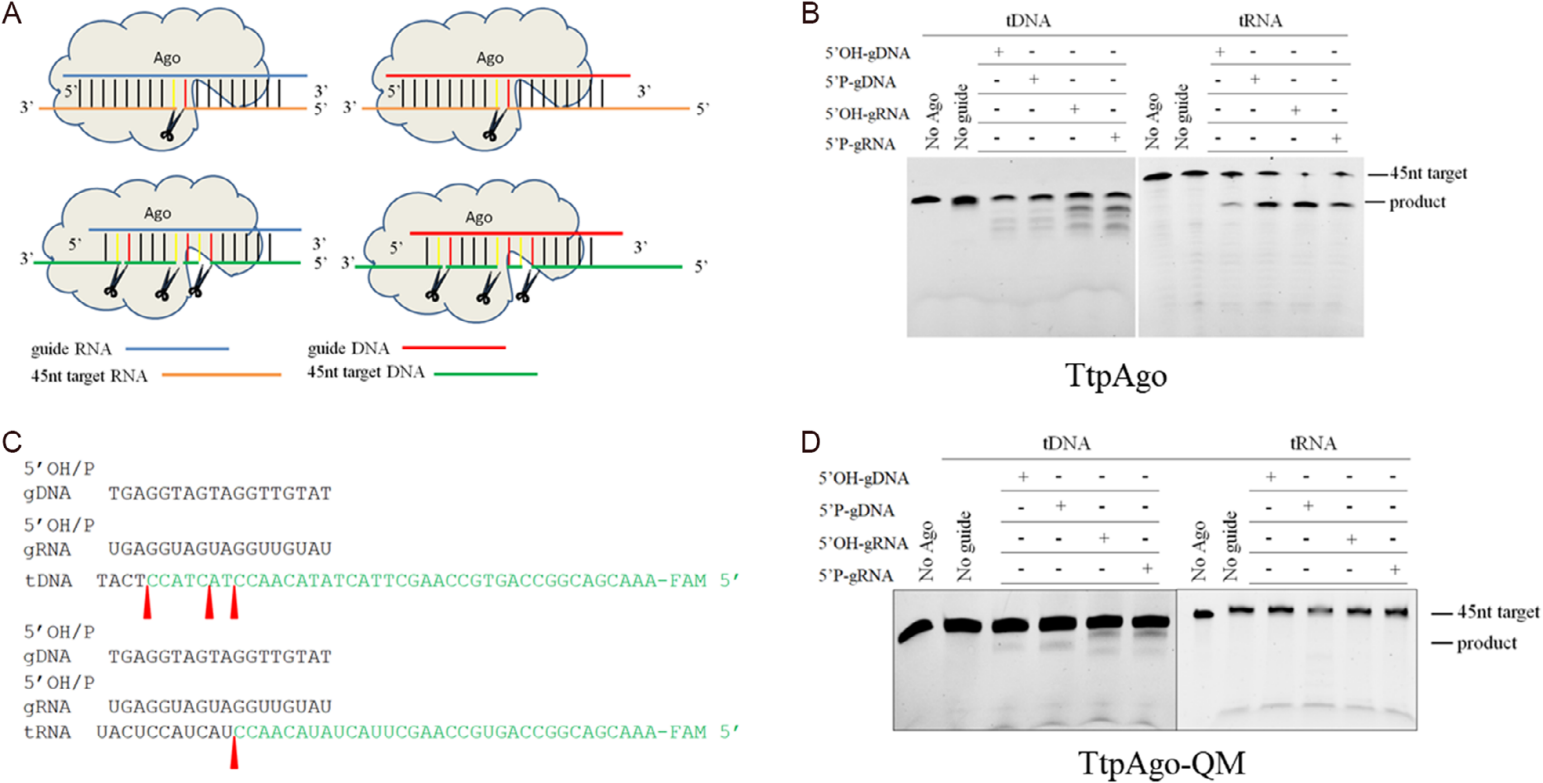
TtpAgo exhibits DNA‐guided and RNA‐guided RNA and DNA cleavage activity in vitro. (A) Cartoon scheme of the guide and target nucleic acids. (B) Cleavage activity assay with FAM‐labeled DNA target and RNA target. (C) Scheme of the guide and target nucleic acids (DNA or RNA); the cleavage position is indicated with a red triangle. (D) Substitutions of four out of four catalytic tetrad residues causes the partially loss of TtpAgo activity.

Nucleic acids specificity of TtpAgo was examined using synthetic, fluorescently labeled oligonucleotide targets, as illustrated in the guide and target nucleic acid diagram (Figure 1A). We tested guide DNA or RNA of 18 nt length, including 5′P and 5′OH‐guides, to cleave ssDNA and RNA targets. TtpAgo demonstrated activity with all eight combinations of guide and target molecules. No cleavage was observed in the absence of guides (Figure 1B). Surprisingly, TtpAgo exhibited DNA cleavage activity, an activity not previously observed for other eAgos [25, 26, 42]. When TtpAgo used gDNAs or gRNAs to cleave DNA targets, it produced multiple cleavage products with sites distinct from the canonical site, located between nucleotides 3–11 from the 5′end of guides. In contrast, when using gDNAs or gRNAs to cleave complementary RNA targets, cleavage occurred at the canonical site between nucleotides 10 and 11 from the 5′end of guides (Figures 1C and S1H, I). We further assessed the dsDNA (double‐stranded DNA) cleavage activity of TtpAgo using supercoiled plasmid DNA (pUC19) (Figure S1J). TtpAgo exhibited only background‐level, guide‐independent degradation of dsDNA, similar to observations reported for some pAgos [11, 27]. In contrast, no degradation of ssDNA was detected in the absence of guides or enzyme (Figure S1K), indicating that this nonspecific activity is restricted to dsDNA. Cleavage of TtpAgo required an intact catalytic tetrad in the PIWI domain. Mutating of the catalytic tetrad residues led to complete abolition of RNA cleavage activity (Figure 1D). However, DNA cleavage activity was reduced but not completely abolished in the PIWI mutants. This observation suggests that the catalytic residues responsible for DNA cleavage in TtpAgo may not be fully captured by the canonical DEDD motif identified by sequence alignment. Indeed, variations in catalytic motifs have been reported for other Agos (e.g., MpAgo contains a DEDN motif) [19], and the precise catalytic configuration of TtpAgo remains unclear. Given the limited resolution of sequence‐based predictions and the lack of high‐resolution structural information, further studies will be required to accurately identify the residues involved in DNA cleavage. In summary, preliminary validation indicates that TtpAgo can utilize all four types of guides (i.e., 5′P‐gDNA, 5′OH‐gDNA, 5′P‐gRNA, and 5′OH‐gRNA) to cleave DNA and RNA in vitro.

### 2.2 Effects of Guide Sequence on TtpAgo

**FIGURE 2:**
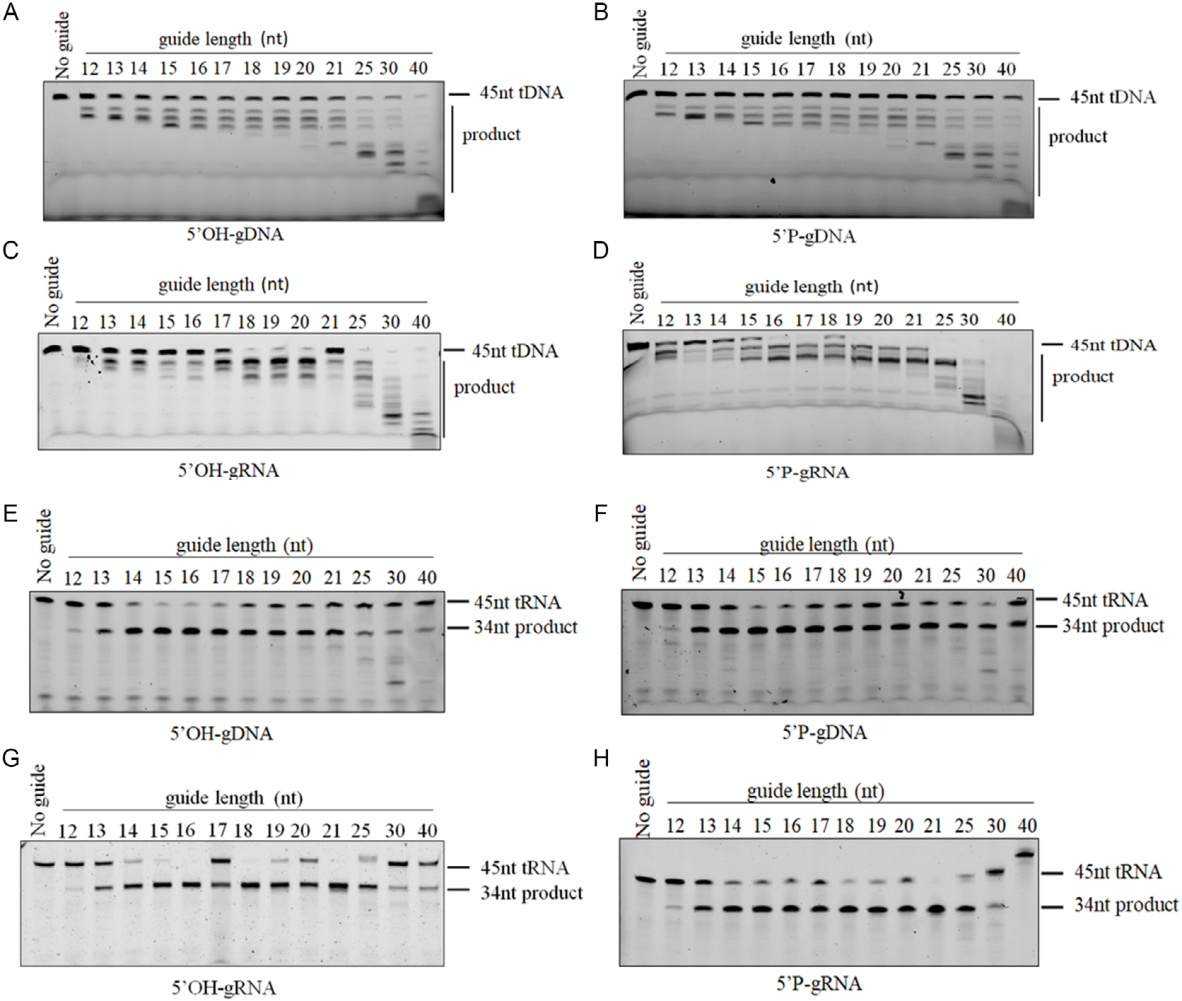
Effects of the guide DNA/RNA length on TtpAgo activity in vitro. (A) Effects of the 5′OH‐gDNA length on DNA cleavage activity. (B) Effects of the 5′P‐gDNA length on DNA cleavage activity. (C) Effects of the 5′OH‐gRNA length on DNA cleavage activity. (D) Effects of the 5′P‐gRNA length on DNA cleavage activity. (E) Effects of the 5′OH‐gDNA length on RNA cleavage activity. (F) Effects of the 5′P‐gDNA length on RNA cleavage activity. (G) Effects of the 5′OH‐gRNA length on RNA cleavage activity. (H) Effects of the 5′P‐gRNA length on RNA cleavage activity.

Most pAgos exhibit functionality across a wide range of guide length [14, 17, 44]. Most eAgos reported use gRNAs of 12–23 nt in length [6, 23], except for human Ago3, which shows a preference for short gRNAs, exhibiting the highest cleavage efficiency with a 14 nt gRNA [26, 45]. We tested the guide length preference of TtpAgo in vitro. Guides ranging from 12–40 nt in length were employed for this assessment in vitro. TtpAgo demonstrated activity across all selected length of gDNAs in the evaluation of 5′OH‐gDNA and 5′P‐gDNA mediated ssDNA cleavage, with cleavage product patterns varying with the length of the gDNA (Figures 2A,B, and S2A,B). Optimal activity was observed when longer gDNAs were used as guides, albeit yielding multiple distinct products. Similarly, utilizing RNA as a guide for targeted DNA cleavage resulted in the generation of multiple cleavage products, with higher efficiency noted when longer gRNAs were employed (Figures 2C,D, and S2C, H). Surprisingly, the target degradation pattern of DNA completely disagrees with conventional target cleavage. When using longer guides, the cleavage position shifts closer to the FAM label, resulting in shorter products. This is unexpected given that Agos typically anchor the 5′end of the guide and cleave specifically between target nucleotides 10 and 11 (i.e. longer guides should still result in cleavage at the same position). This implies that there is no specific cleavage, or that the 3′end of the guide is anchored rather than the 5′end, when targeting and cleaving DNA (i.e. measuring from the 3′end of the guide rather than from the 5′end). To address the unconventional cleavage pattern, we specifically designed experiments using 3′end‐anchored guides with 5′end‐extensions, which revealed that both RNA and DNA guides generated precisely sized DNA cleavage products (Figure S2I and J, etc.), conclusively demonstrating that TtpAgo likely employs a 3′end anchoring mechanism rather than the canonical 5′end guidance, thereby explaining the observed shift in cleavage positions relative to the FAM label when using longer guides.

For 5′OH‐gDNA and 5′P‐gDNA mediated RNA cleavage, TtpAgo exhibited peak cleavage efficiency with gDNAs ranging from 15 to 16 nt in length, showing weaker activity with shorter gDNAs (Figures 2E,F and S2E,F). In the context of 5′OH‐gRNA and 5′P‐gRNA mediated RNA cleavage, the use of a 21 nt gRNA demonstrated efficient cleavage activity, whereas lower activity was observed with shorter gRNAs. Employing a 40 nt 5′P‐gRNA resulted in no detectable RNA cleavage activity (Figures 2G,H and S2G,D). Notably, when cleaving RNA, TtpAgo consistently targeted the conventional cleavage site across different guide length.

**FIGURE 3:**
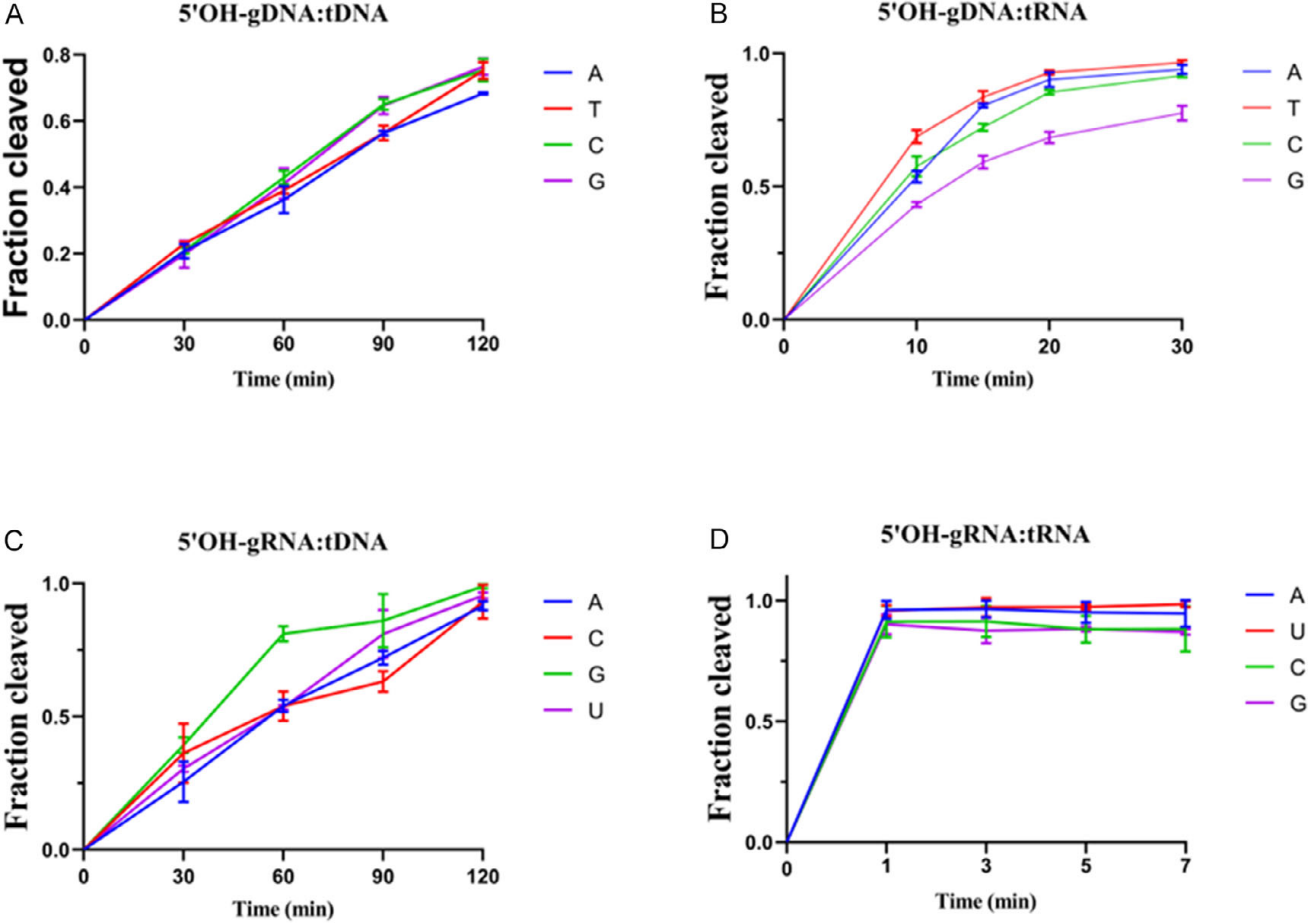
Effects of the 5′end nucleotide identity on TtpAgo activity in vitro. (A) Effects of the 5′end nucleotide of 5′OH‐gDNA on DNA cleavage activity. (B) Effects of the 5′end nucleotide of 5′OH‐gDNA on RNA cleavage activity. (C) Effects of the 5′end nucleotide of 5′OH‐gRNA on DNA cleavage activity. (D) Effects of the 5′end nucleotide of 5′OH‐gRNA on RNA cleavage activity. Error bars represent SD of three independent experiments.

To determine whether TtpAgo has a preference for the first nucleotide of the guide nucleic acid in vitro, in addition to the core sequence, we systematically evaluated four guide RNA and DNA variants that differed exclusively in their 5′terminal nucleotide composition (Table S1). We first systematically assessed the cleavage efficiency of TtpAgo on 45 nt DNA and RNA targets using four 5′OH‐gDNA variants. With DNA targets, the enzyme exhibited no significant base preference at the guide's 5′terminus, demonstrating comparable cleavage efficiencies across all variants (Figures 3A and S3A). Strikingly, when processing RNA targets, the 5′‐G containing gDNA showed reduced catalytic activity compared to 5′‐A, 5′‐T and 5′‐C variants (Figures 3B and S3B). Subsequent parallel experiments using 5′ OH‐gRNA showed that TtpAgo showed higher cleavage efficiency in DNA within 60 min when the 5′‐terminal nucleotide was G, while RNA had no significant preference (Figures 3C,D and S3C,D). These findings suggest that 5′terminal base specificity occurs only in the context of 5′OH‐gDNA‐mediated RNA target cleavage and 5′OH‐gRNA‐mediated DNA, where the efficiency of 5′‐G decreases slightly when targeting RNA and increases when targeting DNA, possibly due to different types of guides, leading to a preference.

### 2.3 Effects of Temperature on the Cleavage Activity of TtpAgo In Vitro

**FIGURE 4:**
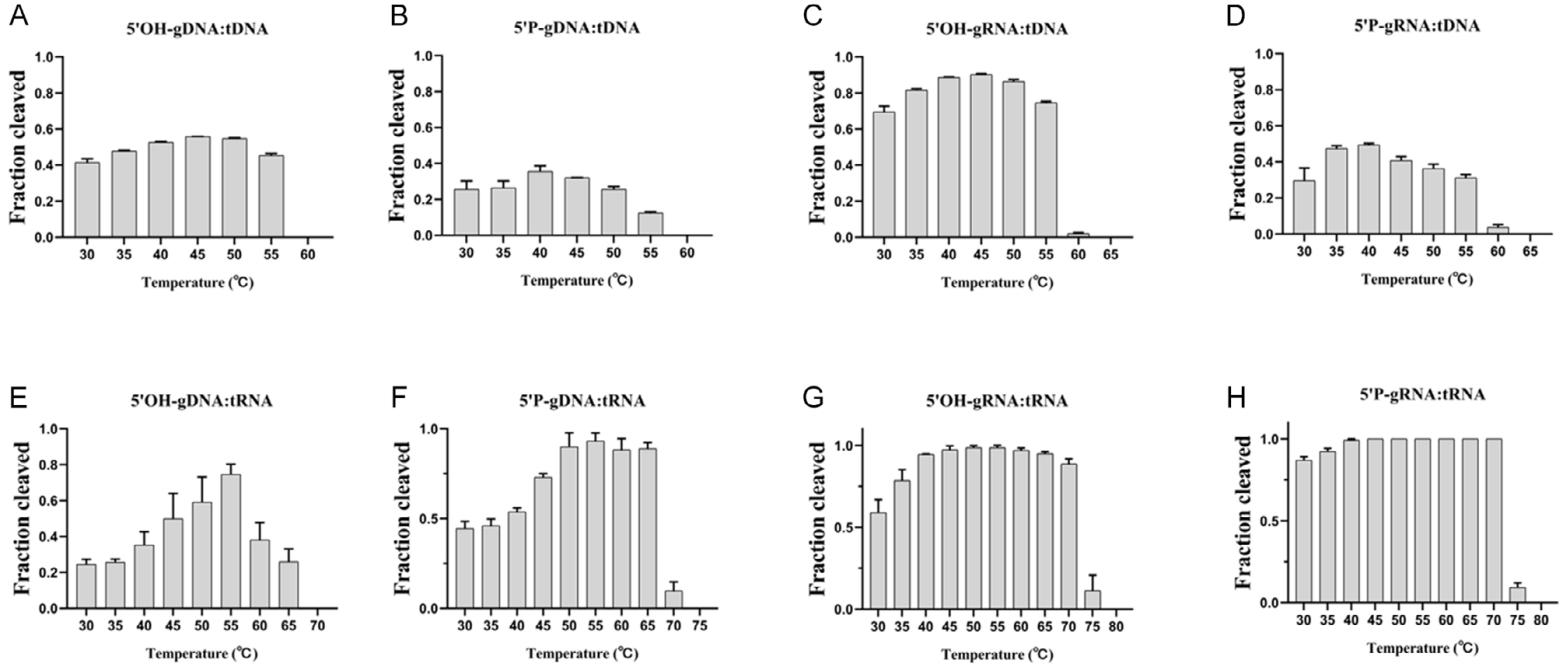
Effects of temperature on TtpAgo activity in vitro. (A) Effects of temperature on DNA cleavage activity mediated by 5′OH‐gDNA. (B) Effects of temperature on DNA cleavage activity mediated by 5′P‐gDNA. (C) Effects of temperature on DNA activity mediated by 5′OH‐gRNA. (D) Effects of temperature on DNA activity mediated by and 5′P‐gRNA. (E) Effects of temperature on RNA activity mediated by 5′OH‐gDNA. (F) Effects of temperature on RNA activity mediated by 5′P‐gDNA. (G) Effects of temperature on RNA activity mediated by 5′OH‐gRNA. (H) Effects of temperature on RNA activity mediated by 5′P‐gRNA. Error bars represent SD of three independent experiments.

To determine the optimal temperature for TtpAgo activity in vitro, a series of cleavage assessments was conducted over a temperature range of 30°C to 85°C. When utilizing 5′OH‐gDNA and 5′P‐gDNA for ssDNA cleavage, TtpAgo exhibited cleavage activity within the range of 30°C–55°C. The optimum temperature for 5′OH‐gDNA cleavage was identified as 45°C, while for 5′P‐gDNA, it was 40°C (Figures 4A,B and S4A,S4B). Similarly, when ssDNA was targeted with 5′OH‐gRNA and 5′P‐gRNA, effective cleavage occurred between 30°C and 60°C; the optimal cleavage temperature for 5′OH‐gRNA was also 45°C, and for 5′P‐gRNA, it was 40°C (Figures 4C,D and S4C,S4D). Notably, cleavage activity decreased significantly at 60°C. In RNA cleavage using 5′OH‐gDNA, 5′P‐gDNA, 5′OH‐gRNA, and 5′P‐gRNA, TtpAgo demonstrated a broader range of effective activity compared to ssDNA cleavage. The highest cleavage efficiency among the four guide types was observed at 55°C. However, the activity of 5′OH‐gDNA decreased sharply at 65°C, 5′P‐gDNA at 70°C, and both 5′OH‐gRNA and 5′P‐gRNA at 75°C (Figures 4E–H and S4E‐H). Figure 4H indicates that when using 5′P‐gRNA as the guide targeting RNA, the cleavage efficiency reaches 100% within the temperature range of 45–70°C; however, the specific temperature for optimal cleavage efficiency remains undetermined. Subsequently, we halved the concentration of TtpAgo and repeated the experiment, ultimately determining that the optimal temperature for cleavage was 55°C (Figure S4I,J). The optimal temperature of DNA cleavage and RNA cleavage differed, with DNA being 40–45°C and RNA being 55°C in vitro.

### 2.4 Effects of Mg^2+^ Concentration on the Cleavage Activity of TtpAgo In Vitro

**FIGURE 5:**
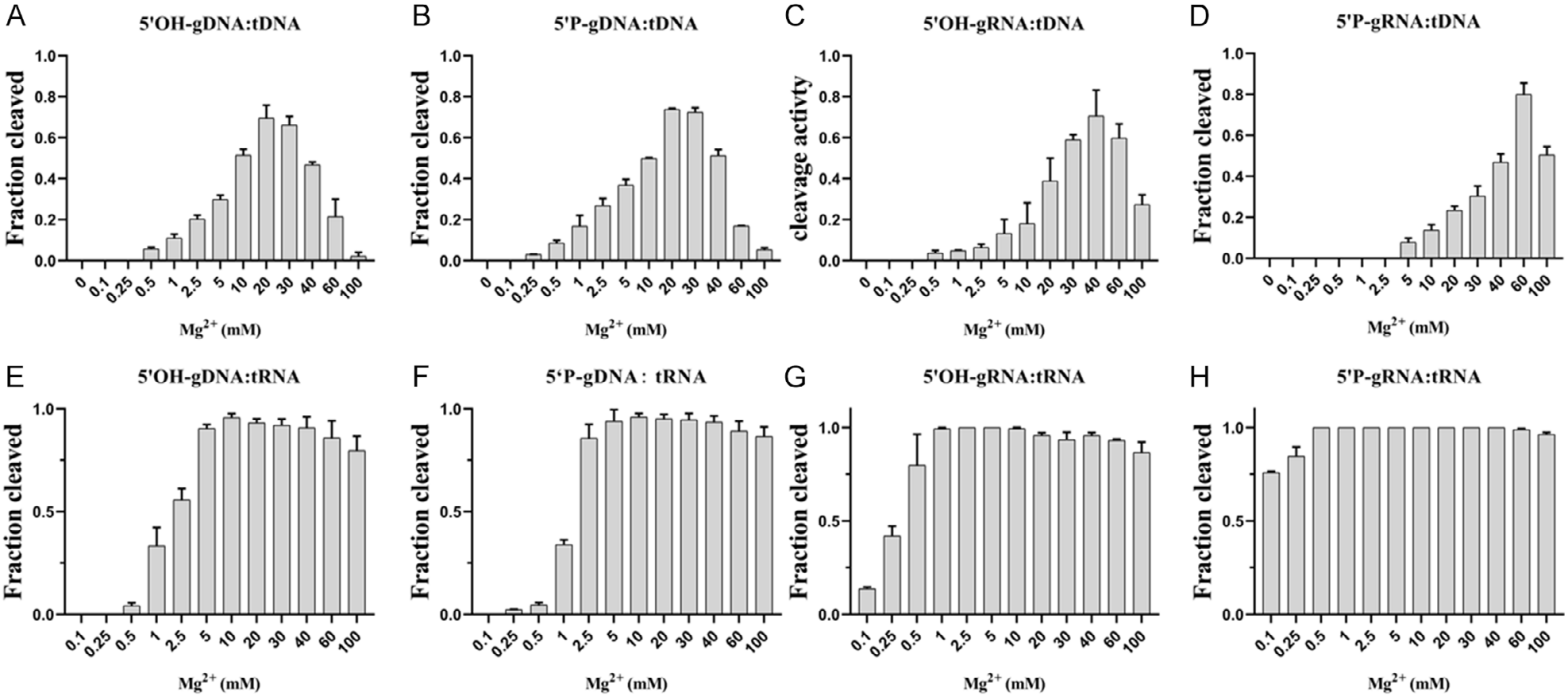
Effects of Mg^2+^ concentration on TtpAgo cleavage activity in vitro. (A) Effects of Mg^2+^ ions concentration on DNA cleavage activity mediated by 5′OH‐gDNA. (B) Effects of Mg^2+^ ions concentration on DNA cleavage activity mediated by 5′P‐gDNA. (C) Effects of Mg^2+^ ions concentration on DNA cleavage activity mediated by 5′OH‐gRNA. (D) Effects of Mg^2+^ ions concentration on DNA cleavage activity mediated by 5′P‐gRNA. (E) Effects of Mg^2+^ ions concentration on RNA cleavage activity mediated by 5′OH‐gDNA. (F) Effects of Mg^2+^ ions concentration on RNA cleavage activity mediated by 5′P‐gDNA. (G) Effects of Mg^2+^ ions concentration on RNA cleavage activity mediated by 5′OH‐gRNA. (H) Effects of Mg^2+^ ions concentration on RNA cleavage activity mediated by 5′P‐gRNA. Error bars represent SD of three independent experiments.

Considering the greatly impact of divalent metal ions on Agos’ activity, we conducted an evaluation of the effects of various divalent metal ions on the cleavage activity of ssDNA and RNA in vitro. Our findings revealed that TtpAgo exhibited cleavage activity primarily in the presence of Mg^2+^ and Mn^2+^ (Figure S5I‐K). Subsequently, we investigated the influence of Mg^2+^ concentration on the cleavage of ssDNA and RNA. Interestingly, we observed that the concentration of Mg^2+^ required for efficient DNA cleavage by TtpAgo was higher than that needed for RNA cleavage. Specifically, the optimal Mg^2+^ concentration for DNA cleavage fell within the range of 20–60 mM (Figures 5A–D and S5A–D), whereas for RNA cleavage, TtpAgo demonstrated optimal activity with Mg^2+^ concentrations between 5 mM and 10 mM (Figures 5E–H and S5E‐H). Considering that high concentrations of Mg^2+^ can cause DNA breakdown, we conducted a negative experiment with only high concentrations of Mg^2+^ and target DNA without adding Ago. The results showed that high concentrations of Mg^2+^ did not result in ssDNA breakage (Figure S5L). Notably, effective cleavage with 5′OH‐gRNA and 5′P‐gRNA could still be achieved at concentrations as low as 0.1 mM Mg^2+^. As with temperature experiments, RNA cleavage efficiency was 100% at a certain concentration range of Mg^2+^ when utilizing 5′P‐gRNA. Next, we also reduced the TtpAgo concentration to half of the original and found that the optimal concentration was 1–2.5 mM (Figure S5M, N). When DNA cleavage is performed in vitro, the concentration of magnesium ions required for TtpAgo is higher than that required to cleave RNA.

### 2.5 Effects of Guide‐Target Mismatches on TtpAgo Cleavage Activity In Vitro

Previous studies have established that single‐base mismatches between the guide and the target can significantly impact the cleavage activity of Agos [14, 17, 23, 44]. Early studies on Agos shown that mismatches in the seed region (g2‐g8) of the guide sequence typically reduce their activity, while mismatches at the 3′supplementary have little impact on activity [46]. Recent studies on several mesophilic pAgos (such as CbAgo [44], LrAgo [44], and KmAgo [14, 15]) found that mismatches in the seed region do not significantly affect the cleavage of target DNA and may even stimulate cleavage, whereas mismatches at the 3′supplementary strongly inhibit activity.

**FIGURE 6:**
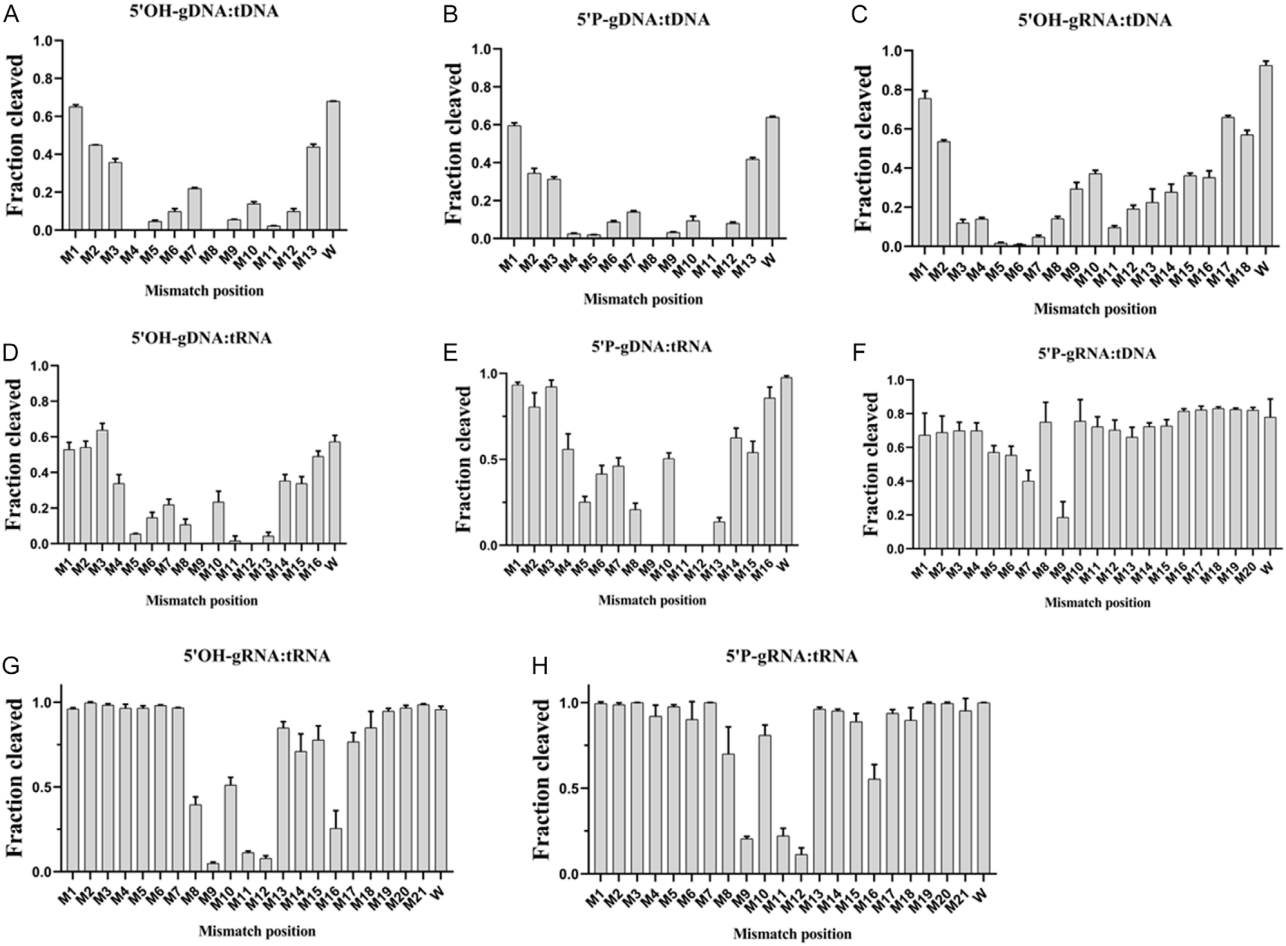
Effects of mismatch position on TtpAgo activity in vitro. (A) 5′OH‐gDNA:tDNA was performed at the 4:1:1 TtpAgo:guide:target molar ratio in reaction buffer containing Mg^2+^ for 120 min at 45°C. (B) 5′P‐gDNA:tDNA was performed at the 4:1:1 TtpAgo:guide:target molar ratio in reaction buffer containing Mg^2+^ for 120 min at 40°C. (C) 5′OH‐gRNA:tDNA were performed at the 4:1:1 TtpAgo:guide:target molar ratio in reaction buffer containing Mg^2+^ for 120 min at 45°C. (D) 5′OH‐gDNA :tRNA was performed at the 4:1:1 TtpAgo:guide:target molar ratio in reaction buffer containing Mg^2+^ for 30 min at 55°C. (E) 5′P‐gDNA :tRNA was performed at the 4:1:1 TtpAgo:guide:target molar ratio in reaction buffer containing Mg^2+^ ions for 20 min at 55°C. (F) 5′P‐gRNA:tDNA were performed at the 4:1:1 TtpAgo:guide:target molar ratio in reaction buffer containing Mg^2+^ for 120 min at 40°C. (G) and (H) 5′OH‐gRNA:tRNA and 5′P‐gRNA:tRNA were performed at the 4:1:1 TtpAgo:guide:target molar ratio in reaction buffer containing Mg^2+^ for 1 min at 55°C. Error bars represent SD of three independent experiments. W, no mismatch.

In contrast to these studies, the effect of mismatches between the guide molecule and the target molecule on TtpAgo appears unusual in vitro. In the DNA‐guided cleavage reaction system (Figures 6A,B,D,E and S6A–D), both 5′P‐gDNA and 5′OH‐gDNA exhibit a high sensitivity to single nucleotide mismatches, with mismatches in the seed region, the region near the cleavage site, and the 3′‐supplementary all leading to a significant decrease in cleavage activity (a reduction of 60%–99%). In comparison, the RNA‐guided system shows differentiated characteristics: except for the sensitivity to mismatches when targeting DNA with 5′OH‐gRNA, which is comparable to that of gDNA (Figures 6C and S6E), other combinations (5′P‐gRNA:tDNA, 5′P‐gRNA:tRNA, 5′OH‐gRNA:tRNA) demonstrate strong tolerance to mismatches at most sites (activity change <10%) (Figures 6F–H and S6F‐H), but a specific decrease in activity occurs when a mismatch is introduced at position 9 (>70%). These distinctive characteristics suggest TtpAgo may employ a novel target recognition mechanism diverging from other Ago paradigms.

Furthermore, previous structural analyses of Cas9 bound to mismatched DNA substrates have demonstrated that insufficient interactions between the gRNA‐tDNA heteroduplex frequently result in site‐specific mismatch tolerance, a phenomenon termed ‘blind spots’ with critical implications for off‐target effects in genome editing applications [47]. In contrast to Cas9, TtpAgo exhibits pronounced sensitivity to single‐base mismatches during DNA‐guided targeting of DNA or RNA substrates, while displaying a complete absence of cleavage activity at specific recognition sites in vitro.

### 2.6 TtpAgo Can use 5′P‐gDNAs and 5′OH/P‐gRNAs to Cleave Highly Structured RNA

Considering TtpAgo's efficient cleavage of RNA guided by both 5′P‐gDNAs and 5′OH/P‐gRNAs, we conducted further exploration into whether TtpAgo‐guide complexes could target and cleave sequences within highly structured RNA molecules characterized by multiple conformational features, such as helices, protrusions, hairpin loops, and various‐length single‐stranded regions in vitro. Dayeh et al. utilized SHAPE (Selective 2′‐Hydroxyl Acylation analyzed by Primer Extension) methodology to predict the secondary structure of the HIV‐1 ΔDIS 5′UTR and designed a set of gDNAs spanning this sequence in increments of 23 nt [23]. The optimal length of TtpAgo‐mediated RNA cleavage for gDNAs and gRNAs were determined to be 16 nt and 21 nt, respectively.

**FIGURE 7:**
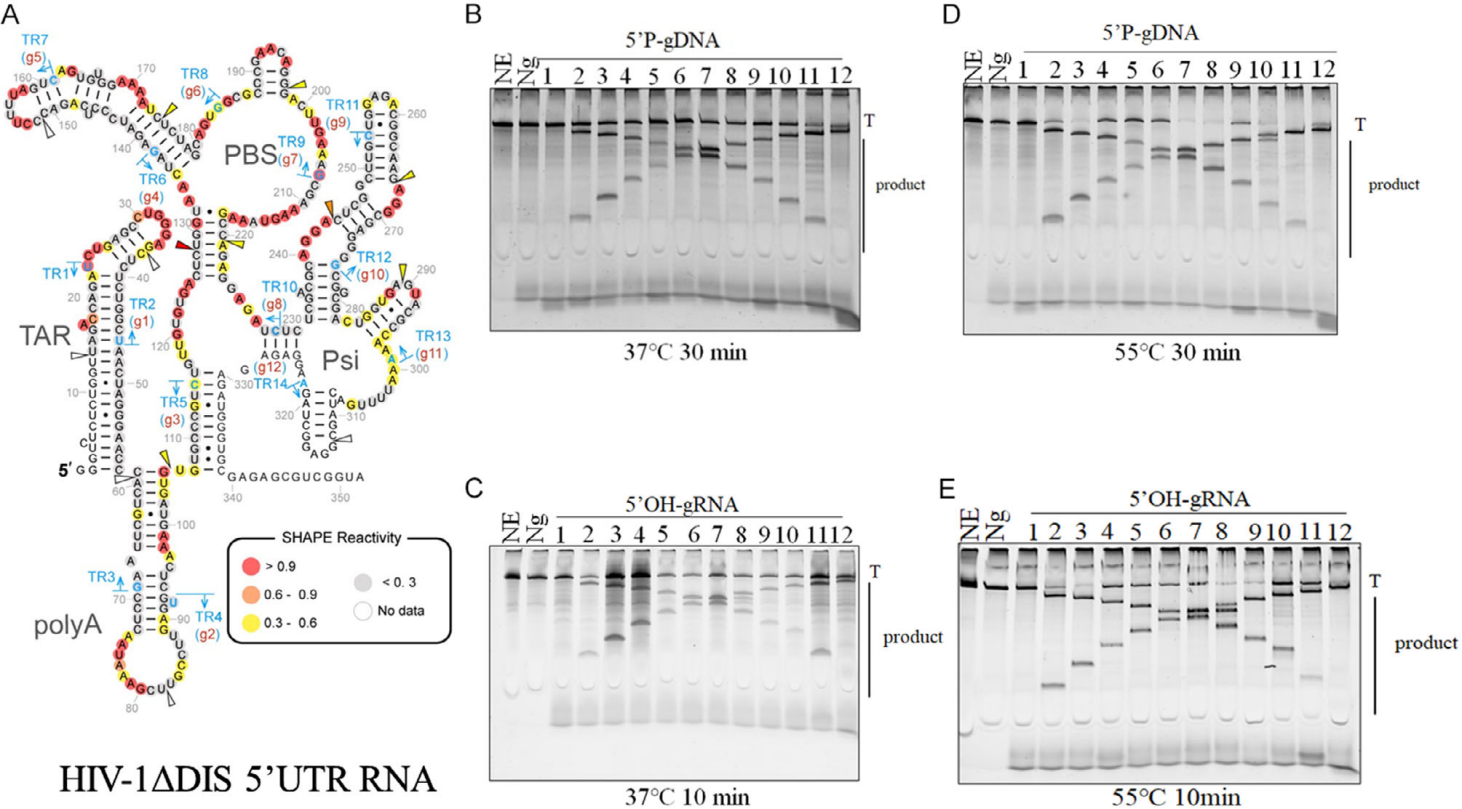
Cleavage activity of TtpAgo complex for highly RNA structures. (A) Secondary structure of HIV‐1 ΔDIS 5′UTR predicted by SHAPE. Colored circles at nucleotide positions indicate SHAPE reactivity. guide DNAs/RNAs targeting the different TRs (target regions) of the HIV transcript are indicated by light blue bars and arrows (TR1 – TR14). Arrowheads indicate cleavage sites by TtpAgo (This figure has been officially licensed, with license number 6125310546169 by the Nucleic Acids Research journal). (B) Substrates and products generated by the assay described in (A) were resolved by denaturing PAGE (8%) revealing cleavability of the highly structured RNA by TtpAgo–gDNA complex in 37°C. (C) Substrates and products generated by the assay described in (A) were resolved by denaturing PAGE (8%) revealing cleavability of the highly structured RNA by TtpAgo–gRNA complex in 37°C. (D) Substrates and products generated by the assay described in (A) were resolved by denaturing PAGE (8%) revealing cleavability of the highly structured RNA by TtpAgo–gDNA complex in 55°C. (E) Substrates and products generated by the assay described in (A) were resolved by denaturing PAGE (8%) revealing cleavability of the highly structured RNA by TtpAgo–gRNA complex in 55°C. T, Target RNA; NE, no enzyme; Ng, no guide.

To assess TtpAgo's cleavage capability against highly structured RNA, we designed 12 gDNAs (16 nt, Figures 7A, S7A and Table S3) and 12 gRNAs (21 nt, Table S3) matching the target sites identified by Dayeh et al. Cleavage products were detected for most of the 5′P‐gDNAs, 5′P‐gRNAs and 5′OH‐gRNAs at temperatures of 37°C and 55°C, respectively; however, no cleavage was observed for gDNA‐1 and gRNA‐1, which were located in a highly stabilized stem region (Figures 7B–E and S7B, 7C). These findings indicated that TtpAgo, like KpAgo [23], KmAgo [14], and MbpAgo [17], can effectively cleave RNA with intricate structures in vitro, with the cleavage efficiency influenced by the RNA's structural characteristics. Notably, the *Staphylococcus aureus* Cas9—a PAM‐independent Cas‐derived RNase—displays strict substrate selectivity, exclusively cleaving unstructured solvent‐accessible regions within target RNAs [48]. In contrast, the PAM‐independent programmable endoribonuclease Cas13a requires only a 56 nt CRISPR RNA (crRNA) for target recognition and cleavage. However, Cas13a remains constitutively active posttarget engagement, exhibiting nonspecific cleavage of bystander single‐stranded RNAs (ssRNAs) through a collateral activity mechanism [49, 50]. TtpAgo effectively circumvents these constraints by enabling systematic profiling of endonucleolytic cleavage sites within long‐structured RNA molecules under in vitro conditions. Overall, these results underscore the potential of TtpAgo as a versatile RNA‐targeting enzyme for in vitro applications.

## 3 Discussion

Previously, individual eAgo nucleases were shown to have strict specificity for RNA targets, such as hAgo2[6], hAgo3[26], and KpAgo [23]. Recently, our group reported CsAgo can utilize both gDNAs and gRNAs for the cleavage of DNA and RNA. However, DNA cleavage activity mediated by gDNA is minimal, and no cleavage activity mediated by 5′OH‐gDNA was detected at temperatures between 35 and 45°C [38]. In contrast, TtpAgo can utilize both 5′P‐gDNA, 5′OH‐gDNA, 5′P‐gRNA, and 5′OH‐gRNA to cleave DNA and RNA targets at temperatures ranging from 30 to 55°C. Thus, TtpAgo represents the first eAgo protein capable of utilizing all combinations of nucleic acids as guides and targets at mesophilic temperatures. Additionally, our findings demonstrate that TtpAgo can target ssDNA and RNA across various reaction conditions, with cleavage efficiency and precision influenced by factors such as temperature, guide nucleic acid length and phosphorylation, as well as complementarity to the target sequence. The primary distinction between TtpAgo and the biochemically characterized eAgos and pAgos is that TtpAgo may anchor the guide strand at the 3′end during DNA cleavage, leading to multiple cleavage products of varying sizes. In contrast, when cleaving RNA, TtpAgo anchors the guide strand at the 5′end, similar to other Agos, and cleaves the target between the 10th and 11th positions of the guide strand, resulting in a single cleavage product.

TtpAgo's ability to target both DNA and RNA with various guides prompted us to examine its structural features. Notably, a recent study [51] on eAgos identified conserved structural regions that specifically interact with the 2′‐OH group of the ribose backbone in RNA guides—a feature proposed to contribute to discriminating between RNA and DNA guides. Taken together, conserved modifications in the eAgo nucleic acid interface are located primarily in the distal half of the guide‐binding region. These changes introduce a positive electrostatic character in this area, which likely plays a role in the selective recognition of RNA guides. Using AlphaFold 3, we generated models of TtpAgo bound to different guide‐target combinations. These models suggest that the N‐terminal domain of TtpAgo adopts a wedge‐like conformation, which appears to pry open the guide‐target pairing at the guide's 3′ end. This feature resembles the N‐terminal arrangement observed in TtAgo, KmAgo, and CbAgo when using gDNAs, but differs from the packing‐like N‐terminal domains of hAgo2 and RsAgo, which interact with gRNAs and targets in a distinct manner (Figure S9A, Figure S10A‐D). We also observed variability in how TtpAgo interacts with the 3′end of the guide, which may influence pairing with target nucleic acids. In our models, DNA targets pair with guide nucleotides 2–18, whereas RNA targets pair with nucleotides 2–17 (Figure S9B). This difference may contribute to the slight shift in cleavage position when TtpAgo acts on DNA versus RNA targets: cleavage occurs between nucleotides 10–11 from the guide 5′end for RNA, while DNA cleavage is offset. By contrast, TtAgo and KmAgo exhibit consistent structural arrangements and cleavage positions for both DNA and RNA targets (Figure S10B, C). We emphasize that these conclusions are derived from computational models, which provide structural insights but cannot definitively predict guide/target binding or cleavage specificity. Experimental validation, such as mutagenesis or biochemical assays, will be required to confirm these observations.

The diverse catalytic activities exhibited by TtpAgo (including RNA/DNA dual substrate recognition) suggest that its physiological functions require further investigation. Analysis of copurified nucleic acids indicates that TtpAgo expressed in *E. coli* and purified through sequential Ni‐NTA, heparin affinity, and size‐exclusion chromatography predominantly associates with small RNAs. In contrast, preparations obtained after Ni‐NTA purification alone contained both small RNAs (<40 nt) and high‐molecular‐weight DNA (>100 nt); however, the latter is most likely attributable to nonspecific binding of DNA to the Ni‐NTA resin and its coelution with the target protein. Notably, as TtpAgo originates from a thermophilic fungus, the use of a mesophilic *E. coli* expression system may influence guide composition. The lower growth temperature (37°C versus 50–60°C in the native host) and the absence of native auxiliary factors could affect nucleic acid processing and guide loading. Future studies employing thermophilic expression systems will be necessary to more accurately define the nature of TtpAgo‐associated guide strands under native‐like conditions.

As a novel type of programmable eAgo nuclease, TtpAgo exhibits multiple application values: it can achieve RNA target cleavage (including highly‐structured RNA) guided by DNA/RNA at physiological temperatures. This characteristic supports the development of transfection complexes similar to the hAgo2‐siRNA system for RNA knockdown in mammalian cells [52], and due to its distant evolutionary relationship with hAgo2 (identity = 31.97%), it may have lower toxicity. Notably, its differentiated cleavage ability on RNA stem‐loop regions provides a new tool for high‐throughput analysis of RNA secondary structures. Although the DNA editing application of TtpAgo is limited by its guide‐independent dsDNA degradation activity—potentially due to endogenous nucleic acid contamination introduced during purification—genome editing may still be achievable. This could be accomplished by optimizing purification protocols to remove possible endogenous nucleic acids, followed by transfection of either the preassembled TtpAgo–guide complex or a plasmid encoding both TtpAgo and the guide DNA/RNA. Additionally, its broad substrate specificity supports the development of novel detection technologies, such as establishing a molecular diagnostic platform similar to KmAgo through combined isothermal amplification [53]. More importantly, it reveals the diversity treasure of eAgos‐existing in almost all eukaryotic species, some with multiple copies [2]‐providing abundant resources for discovering new programmable DNA nucleases.

Evolutionarily, its existence suggests unexplored diversity in eukaryotic gene silencing mechanisms, potentially reflecting ancestral Ago versatility [2]. Our findings redefine the functional scope of eAgos and position this enzyme as a versatile tool for manipulating nucleic acid landscapes in research and therapeutics.

## 4 Methods

### 4.1 Protein Expression and Purification

The TtpAgo gene (XP_006694702.1; *Thermochaetoides thermophila* DSM 1495) and the TtpAgo quadruple mutant (TtpAgo‐QM; D765A/E809A/D843A/D988A) were synthesized by Wuhan Genecreate Biotechnology Co., Ltd and cloned into pET28a expression vectors, ensuring an in‐frame insertion with an N‐terminal 6× His tag. The recombinant TtpAgo and TtpAgo‐QM proteins were expressed in *Escherichia coli* BL21(DE3) (Tsingke). Cultures were grown in Luria‐Bertani (LB) medium supplemented with 50 μg/ml kanamycin at 37°C until the optical density at 600 nm (OD_600_) reached 0.8. Cells underwent cold shock at 0°C for 30 min, followed by induction of TtpAgo expression with the addition of isopropyl‐*β*‐D‐1‐thiogalactopyranoside (IPTG) to a final concentration of 0.5 mM. Expression was carried out at 18°C for 17 h with continuous shaking. The harvested cells were centrifuged and stored at −80°C prior to protein purification.

Cell lysis was performed using high‐pressure homogenization in Buffer A, which consisted of 20 mM HEPES–NaOH (pH 7.5), 250 mM NaCl, and 1 mM phenylmethanesulfonyl fluoride (PMSF). The lysate was clarified by centrifugation at 14,000 rpm for 30 min, and the resulting supernatant underwent purification using Ni‐NTA affinity chromatography. The TtpAgo protein was eluted with Buffer A containing 200 mM imidazole and concentrated to 1 mL using 50 kDa molecular weight cut‐off ultrafiltration tubes. Subsequently, the protein was applied to a Heparin column (HiTrap Heparin HP, GE Healthcare) equilibrated with Buffer B (20 mM HEPES–NaOH, pH 7.5, 200 mM NaCl). The column was washed with at least eight column volumes of the same buffer and eluted using a linear NaCl gradient (0.2–2 M). Fractions containing TtpAgo were concentrated again by ultrafiltration with 50 kDa ultrafiltration tubes. Lastly, the heparin‐purified protein was loaded onto a size exclusion column (Superdex 200 10/300; GE Healthcare) for additional purification. Fractions containing TtpAgo were stored in Buffer A at ‐80°C.

### 4.2 Copurification of Nucleic Acids with TtpAgo and sfGFP

Proteinase K (Ambion) was added to purified TtpAgo or sfGFP at final concentration of 1 mg/mL and the sample was incubated for 1 h at 55°C. The nucleic acids were separated from the organic fraction by adding Roti‐phenol/chloroform/isoamyl alcohol (pH 7.5–8.0) in a 1:1 ratio (V/V) and following centrifugation at 12,000 rpm for 15 min. Transfer the top layer into 99% ethanol at double the volume, thoroughly mix, incubate overnight at ‐20°C, centrifuge at 12,000 rpm for 30 min, and remove the supernatant. Next, the nucleic acid pellet was washed twice with 700 μL of 70% ethanol and solved in 20 μL nuclease‐free water. The purified nucleic acids were treated with either 100 μg/mL RNase A (Thermo Fisher Scientific) or 2 units DNase I (Thermo Fisher Scientific) or both for 30 min at 37°C and then resolved on a denaturing urea polyacrylamide gel (20%), followed by staining with SYBR Gold and imaging by GelDoc Go Imaging System.

### 4.3 Small RNA Analysis

A total input RNA amount of 1 μg per sample was used for library preparation. Sequencing libraries were generated using the Hieff NGS Ultima Dual‐mode mRNA Library Prep Kit for Illumina (Yeasen Biotech, Shanghai, China), with index codes added to attribute sequences to each sample. Libraries were sequenced on an Illumina NovaSeq 6000 platform to generate 150 bp paired‐end reads. Raw fastq data (raw reads) were processed with in‐house Perl scripts to obtain clean reads by removing adapters, poly‐N‐containing reads, and low‐quality sequences. Clean reads were mapped to the reference genome of *E. coli* BL21(DE3) (GenBank: NC_012892.2) using Bowtie2 2.2.4, retaining reads with a perfect match or one mismatch for downstream analysis. Gene function annotation was performed against Nr (NCBI nonredundant protein sequences), Pfam (protein families), and EGGNOG_class_annotation (Evolutionary Genealogy of Genes: Nonsupervised Orthologous Groups class annotation). Ten reads with the highest kurtosis were analyzed using Samtools v1.22.1 and Seqkit v2.0.0.

### 4.4 Single‐Stranded Nucleic Acids Cleavage Assays

Cleavage assays were conducted using synthetic guides and targets, with oligonucleotide sequences listed in Table S1. For specific experiments, 5′‐FAM‐labeled targets and 5′phosphorylated (5′‐P) guides were synthesized. The assays involved mixing 800 nM TtpAgo with 200 nM gDNA or gRNA and incubating the mixture for 5 min at 40°C for guide loading in buffer RB (10 mM HEPES–NaOH, pH 7.5, 100 mM NaCl, and 5% glycerol), along with 10 mM concentrations of MgCl_2_. After loading, target nucleic acids were added to achieve a final concentration of 200 nM. Cleavage reactions were performed in PCR tubes at 45°C and stopped after specified time intervals by mixing the samples with equal volumes of 2 × RNA loading dye [95% formamide, 18 mM EDTA, 0.025% sodium dodecyl sulfate (SDS), and 0.025% bromophenol blue], followed by heating for 5 min at 95°C.

To investigate the effects of various divalent cations on DNA cleavage, reactions included 20 mM of the following ions: Mn^2+^, Mg^2+^, Ni^2+^, Co^2+^, Cu^2+^, Fe^2+^, Ca^2+^, or Zn^2+^, and RNA cleavage reactions incorporated 5 mM of the same ions. For analyzing temperature dependence in DNA cleavage, TtpAgo was loaded with gDNAs or gRNAs at 40°C for 5 min. The samples were then transferred to specified temperatures in a PCR thermocycler (T100, Bio‐Rad), after which DNA targets were added and incubated for 120 min. To investigate the temperature dependence of RNA cleavage, TtpAgo was preloaded with gDNAs or gRNAs at 50°C for 5 min. The samples were then transferred to specific temperatures in the thermocycler (T100, Bio‐Rad). Subsequently, RNA targets were introduced with varying incubation times ranging from 3 to 30 min (gRNA:tRNA for 3 min, 5′P‐gDNA:tRNA for 20 min, 5′OH‐gDNA:tRNA for 30 min). Cleavage products were resolved using 20% denaturing polyacrylamide gel electrophoresis, visualized with GelDoc Go Imaging System, and analyzed using ImageJ and Prism 8 software.

### 4.5 Double‐Stranded Nucleic Acids Cleavage Assays

The process is the same as single‐stranded nucleic acid cleavage, except that after the reaction ends, treatment is done with 1 mg/ml of protease K at 55°C for 15 min. Agarose gel electrophoresis identification was performed after treatment. The assays involved mixing 800 nM TtpAgo with 1000 nM 5′OH‐gRNA (Supplementary Table S1) and incubating the mixture for 5 min at 40°C for guide loading in buffer RB (10 mM HEPES–NaOH, pH 7.5, 100 mM NaCl, and 5% glycerol), along with concentrations of MgCl_2_ 20 mM. After loading, target nucleic acids were added to achieve a final concentration of 200 ng. Cleavage reactions were performed in PCR tubes at 45°C for 10 min.

### 4.6 Highly‐Structured RNA Cleavage Assays

The HIV‐1 ΔDIS 5′‐untranslated region (UTR) RNA was in vitro transcribed using T7 RNA polymerase (Thermo Fisher Scientific) and synthetic DNA templates containing a T7 promoter sequence. Following transcription, the RNA transcripts were treated with DNase I, and precipitated with ethanol. The gDNAs and gRNAs used for cleavage (listed in Table S3) were 5′phosphorylated using T4 polynucleotide kinase (PNK, New England Biolabs), except for experiments utilizing 5′‐OH guides. For cleavage assays, 800 nM TtpAgo was mixed with 200 nM gDNA or gRNA and incubated for 5 min at 50°C to facilitate guide loading. The RNA target was then added to achieve a final concentration of 200 nM. Subsequent cleavage reactions were carried out at either 37°C or 55°C for 10 or 30 min, respectively. Reactions were terminated by the addition of 2 × RNA loading dye, and samples were heated for 5 min at 95°C. Cleavage products were analyzed through 8% urea denaturing polyacrylamide gel electrophoresis and visualized using SYBR Gold staining.

### 4.7 Structural Simulation of TtpAgo

We input TtpAgo's protein, guide DNA/RNA, and target DNA/RNA sequences into Alphafold 3 for prediction. The guide DNA/RNA is 18 nt, and the target sequence is 20 nt, except for the guide DNA, which has a target length of 25 nt, because when the target is 20 nt, the prediction model will use the RNA target as the guide. The sequence is shown in the Table S1.

## Supporting Information

Additional supporting information can be found online in the Supporting Information section. **Supporting Fig. S1:** Purification of TtpAgo protein, bioinformatics analysis, and *in vitro* biochemical activity analysis. (A) Maximum likelihood phylogenetic tree analysis of TtpAgo based on amino acid sequences. The numbers at the nodes indicate the bootstrap values for maximum likelihood analysis of 1000 resampled data sets. (B) The purity of the purified TtpAgo was determined using SDS‐PAGE. (C) The purity of the purified TtpAgo‐QM was determined using SDS‐PAGE. (D) Nucleic acids associated with TtpAgo after heparin affinity and size‐exclusion chromatography purification. Samples were treated with DNase I (D), RNase A (R), both nucleases (DR), or left untreated (‐). (E, F) Nucleic acids associated with TtpAgo (E) or sfGFP (F) after Ni‐NTA purification. Samples were treated with DNase I (D), RNase A (R), both nucleases (DR), or left untreated (‐). (G) Multiple sequence alignment of a part of the PIWI domain from the TtpAgo with several other characterized Ago proteins. (H) Determining the DNA cleavage site with non‐labeled tDNA. DNA marker (34, 35, 36, 37, 38, 39, 40, 41, 42, 43 nt) were partially hydrolyzed tDNA. (I) Determining the RNA cleavage site with FAM‐labeled tRNA. RNA marker (33, 34, 35 nt) were chemically synthesized 5’‐end, FAM‐labeled. (J) Cleavage assays of double‐stranded nucleic acids. (K) Time‐course analysis of ssDNA degradation. NE, no enzyme; Ng, no guide. **Supporting Fig. S2:** Effects of the guide length on **TtpAgo activity.** (A) Effects of the 5’OH‐gDNA length on DNA cleavage activity. (B) Effects of the 5’P‐gDNA length on DNA cleavage activity. (C) Effects of the 5’OH‐gRNA length on DNA cleavage activity. (D) Effects of the 5’P‐gRNA length on RNA cleavage activity. (E) Effects of the 5’OH‐gDNA length on RNA cleavage activity. (F) Effects of the 5’P‐gDNA length on RNA cleavage activity. (G) Effects of the 5’OH‐gRNA length on RNA cleavage activity. (H) Effects of the 5’P‐gRNA length on DNA cleavage activity. Error bars represent SD of three independent experiments. (I) Guide DNA with 3’ end anchoring and 5’ end extension cleaves tDNA. (J) Guide RNA with 3’ end anchoring and 5’ end extension cleaves tDNA. **Supporting Fig. S3: Effects of the 5’‐end nucleotide identity on** **TtpAgo activity.** (A) Effects of the 5’‐end nucleotide of 5’OH‐gDNA on DNA cleavage activity. (B) Effects of the 5’‐end nucleotide of 5’OH‐gDNA on RNA cleavage activity. (C) Effects of the 5’‐end nucleotide of 5’OH‐gRNA on DNA cleavage activity. (D) Effects of the 5’‐end nucleotide of 5’OH‐gRNA on RNA cleavage activity. All experiments were performed at the 4:1:1 TtAgo:guide:target molar ratio in reaction buffer containing Mg^2+^ ions. **Supporting Fig. S4: Effects of temperature on** **TtpAgo activity.** (A) Effects of temperature on DNA cleavage activity mediated by 5’OH‐gDNA. (B) Effects of temperature on DNA cleavage activity mediated by 5’P‐gDNA. (C) Effects of temperature on DNA activity mediated by 5’OH‐gRNA. (D) Effects of temperature on DNA activity mediated by and 5’P‐gRNA. (E) Effects of temperature on RNA activity mediated by 5’OH‐gDNA. (F) Effects of temperature on RNA activity mediated by 5’P‐gDNA. (G) Effects of temperature on RNA activity mediated by 5’OH‐gRNA. (H) Effects of temperature on RNA activity mediated by 5’P‐gRNA. (I) Effects of temperature on RNA activity mediated by 5’P‐gRNA under the condition of halving the TtpAgo concentration. Error bars represent SD of three independent experiments. (J) Gel image of the effect of temperature on 5’P‐gRNA‐mediated RNA activity under the condition of halving the TtpAgo concentration. **Supporting Fig. S5: Effects of Mg^2+^ concentration on** **TtpAgo cleavage activity.** (A) Effects of Mg^2+^ ions concentration on DNA cleavage activity mediated by 5’OH‐gDNA. (B) Effects of Mg^2+^ ions concentration on DNA cleavage activity mediated by 5’P‐gDNA. (C) Effects of Mg^2+^ ions concentration on DNA cleavage activity mediated by 5’OH‐gRNA. (D) Effects of Mg^2+^ ions concentration on DNA cleavage activity mediated by 5’P‐gRNA. (E) Effects of Mg^2+^ ions concentration on RNA cleavage activity mediated by 5’OH‐gDNA. (F) Effects of Mg^2+^ ions concentration on RNA cleavage activity mediated by 5’P‐gDNA. (G) Effects of Mg^2+^ ions concentration on RNA cleavage activity mediated by 5’OH‐gRNA. (H) Effects of Mg^2+^ ions concentration on RNA cleavage activity mediated by 5’P‐gRNA. (I) Effects of different divalent metal ions on RNA cleavage activity mediated by 5’OH‐gRNA. (J) Effects of different divalent metal ions on RNA cleavage activity mediated by 5’OH‐gDNA. (K) Effects of different divalent metal ions on DNA cleavage activity mediated by 5’OH‐gDNA. (L) Effects of different concentrations of magnesium ions on single‐stranded DNA. (M) Gel image of the effect of Mg^2+^ ions concentration on 5’P‐gRNA‐mediated RNA activity under the condition of halving the TtpAgo concentration. (N) Effects of Mg^2+^ ions concentration on RNA activity mediated by 5’P‐gRNA under the condition of halving the TtpAgo concentration. Error bars represent SD of three independent experiments. The assay in (A) was performed in 45°C for 120 min at indicated Mg^2+^ ions concentration. The assay in (B) was performed in 40°C for 120 min at indicated Mg^2+^ ions concentration. The assay in (C) was performed in 45°C for 120 min at indicated Mg^2+^ ions concentration. The assay in (D) was performed in 40°C for 120 min at indicated Mg^2+^ ions concentration. The assay in (E), 5’OH‐gDNA:RNA was performed in 55°C for 30 min at indicated Mg^2+^ ions concentration. The assay in (F), 5’P‐gDNA:RNA was performed in 55°C for 20 min at indicated Mg^2+^ ions concentration. The assay in (G), (H) were performed in 55°C for 3 min at indicated Mg^2+^ ions concentration. **Supporting Fig. S6: Effects of mismatch position on** **TtpAgo activity.** (A) 5’OH‐gDNA:tDNA was performed at the 4:1:1 TtpAgo:guide:target molar ratio in reaction buffer containing Mg^2+^ ions for 120 min at 45°C. (B) 5’P‐gDNA:tDNA was performed at the 4:1:1 TtpAgo:guide:target molar ratio in reaction buffer containing Mg^2+^ ions for 120 min at 40°C. (C) 5’OH‐gDNA:tRNA were performed at the 4:1:1 TtpAgo:guide:target molar ratio in reaction buffer containing Mg^2+^ ions for 30 min at 55°C. (D) 5’P‐gDNA:tRNA was performed at the 4:1:1 TtpAgo:guide:target molar ratio in reaction buffer containing Mg^2+^ ions for 20 min at 55°C. (E) 5’OH‐gRNA:tDNA was performed at the 4:1:1 TtpAgo:guide:target molar ratio in reaction buffer containing Mg^2+^ ions for 120 min at 45°C. (F) 5’P‐gRNA:tDNA were performed at the 4:1:1 TtpAgo:guide:target molar ratio in reaction buffer containing Mg^2+^ ions for 120 min at 40°C. (G) and (H) 5’OH‐gRNA:tRNA and 5’P‐gRNA:tRNA were performed at the 4:1:1 TtpAgo:guide:target molar ratio in reaction buffer containing Mg^2+^ ions for 1 min at 55°C. Error bars represent SD of three independent experiments. W, no mismatch. **Supporting Fig. S7: Cleavage activity of** **TtpAgo complex for highly RNA structures.** (A) Schematic overview of the HIV‐1 ΔDIS 5’UTR. Arrows with different colors indicate the target region and the corresponding gDNAs and gRNAS are numbered from 1 to 12 with the corresponding colours. (B) Substrates and products generated by the assay described in Figure 7A were resolved by denaturing PAGE (8%) revealing cleavability of the highly structured RNA by TtpAgo–gRNA complex in 37°C. (C) Substrates and products generated by the assay described in Figure 7A were resolved by denaturing PAGE (8%) revealing cleavability of the highly structured RNA by TtpAgo–gRNA complex in 55°C. T, Target RNA; NE, no enzyme; Ng, no guide. **Supporting Fig. S8: Multiple sequence alignment of** **TtpAgo with the characterized Agos.** (A) Sequence alignment of some MID domains. (B) Sequence alignment of some PIWI domains. (C) Sequence alignment of some PAZ domains. **Supporting Fig. S9**: TtpAgo structure analysis. (A) (Upper) Schematic representation of TtpAgo domains: N‐terminal (blue), L1 (yellow), PAZ (pink), L2 (gray), MID (orange), and PIWI (green). (Lower panal) Structural models of TtpAgo with different guides and targets. (B) Close‐up view showing only the guide and target strands. **Supporting Fig. S10: Ago structures.** (A) Target recognition model of the packing‐type N‐terminal domain in hAgo2 and RsAgo. (B) Target recognition model of the wedge‐type N‐terminal domain in TtAgo. (C) Target recognition model of the wedge‐type N‐terminal domain in KmAgo. (D) Target recognition model of the wedge‐type N‐terminal domain in CbAgo. **Supporting Table S1:** List of sequence of gDNAs and gRNAs targeting DNA or RNA. **Supporting Table S2:** List of the ten reads with the highest kurtosis. **Supporting Table S3:** List of gDNAs and gRNAs targeting HIV‐1 ΔDIS 5′UTR RNA.

## Author Contributions

**Zhizhao Chen** conceived the data characterization and designed the experiments and wrote the manuscript. **Fei Wang** and **Xiaolan Yu** designed the experiments. **Yang Liu** designed the experiments and wrote the manuscript. **Lixin Ma** provided the concept, resources, funds and supervision. All authors contributed to the work.

## Funding

This study was supported by Project of Technological Innovation Plan in Hubei Province (Grant 2024BCA001), Natural Science Foundation of Wuhan City (Grant 2024040701010046 and 2025041001010348), Science and Technology Program Project of Hubei Province (Grant 2025CSA051), Hubei Provincial Science Fund for Distinguished Young Scholars (Grant 2024AFA101).

## Conflicts of Interest

The authors declare no conflicts of interest.

## Supporting information

Supplementary Material

## Data Availability

The data that support the findings of this study are available in the supplementary material of this article.
