# Supplementary material for: A Programmable Eukaryotic Argonaute Nuclease with Dual DNA and RNA Cleavage Activity from Thermophilic Fungus Thermochaetoides thermophila
Source: Small Sci. 2026 Mar 15;6(3):e202500237. doi: 10.1002/smsc.202500237 (PMC13097340; doi:10.1002/smsc.202500237)
Supplement: Supplementary file 1 — Supplementary Material [file SMSC-6-e202500237-s001.pdf]

Supplementary Materials

A Programmable Eukaryotic Argonaute Nuclease with Dual DNA and RNA Cleavage Activity from Thermophilic Fungus *Thermochaetoides thermophila*

Zhizhao Chen[1], Fei Wang[1], Xiaolan Yu[1], Yang Liu[1,*], Lixin Ma[1,*]

[1] State Key Laboratory of Biocatalysis and Enzyme Engineering, Hubei Key Laboratory of Industrial Biotechnology, School of Life Sciences, Hubei University, Wuhan, Hubei 430062, China

[*] Correspondence should be addressed to YL (lyang@hubu.edu.cn) and LM (malixing@hubu.edu.cn)

Content

Supplementary Figures:

Supplementary Figure S1. Purification of TtpAgo protein, bioinformatics analysis, and in vitro biochemical activity analysis.

Supplementary Figure S2. Effects of the guide length on TtpAgo activity.

Supplementary Figure S3. Effects of the 5'-end nucleotide identity on TtpAgo activity.

Supplementary Figure S4. Effects of temperature on TtpAgo cleavage activity.

Supplementary Figure S5. Effects of Mg^{2+} concentration on TtpAgo cleavage activity.

Supplementary Figure S6. Effects of mismatch position on TtpAgo activity

Supplementary Figure S7. Cleavage activity of TtpAgo complex for highly RNA structures.

Supplementary Figure S8. Multiple sequence alignment of TtpAgo with the characterized Agos.

Supplementary Figure S9. TtpAgo structure analysis.

Supplementary Figure S10. Ago structures.

Supplementary Tables:

Supplementary Table S1. List of sequence of gDNAs and gRNAs targeting DNA or RNA

Supplementary Table S2. List of the ten reads with the highest kurtosis.

Supplementary Table S3. List of sequence of gDNAs and gRNAs targeting HIV-1 ΔDIS 5′UTR RNA

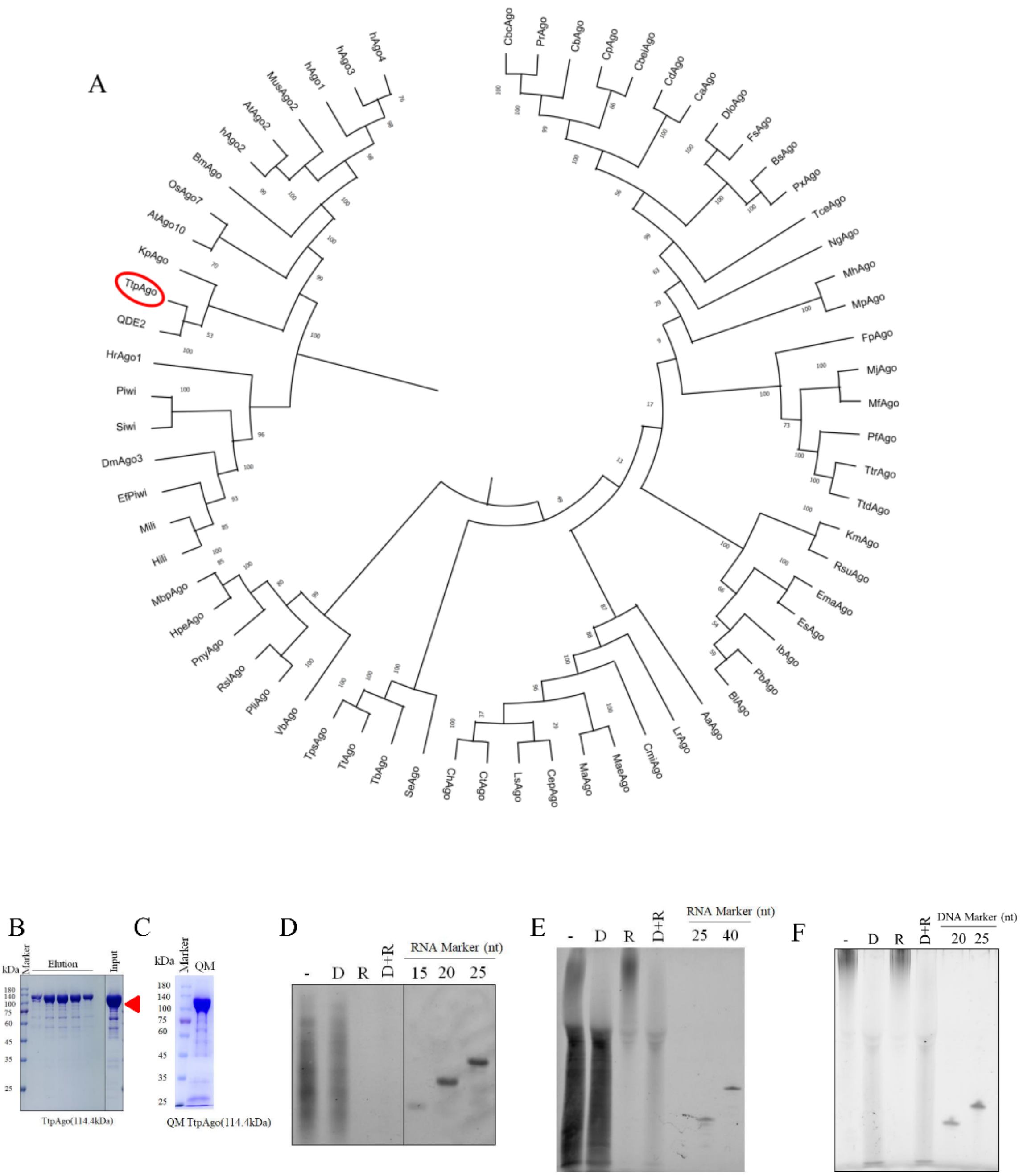

G

Protein	Source	D	E	D	X	Identity(%)
hAgo2	-*Homo sapiens*	FLGADVTHPP	QHRQEIIQDL	IFYRDGVSEQ	AYYAHLVAFR	31.97%
KpAgo	-*Kluyveromyces Ployspours*	VLGSDVTHYP	DGPGEEIITN	MYFRDGVSVD	VYYADLLCTR	33.25%
AtAgo1	-*Arabidopsis thaliana*	IFGADVTHPH	AHRQELIQDL	IFYRDGVSEG	AYYAHLAAFR	27.89%
PfAgo	-*Pyrococcus furiosus*	IIGIDVAPMK	EQRGESVDMN	LLLRDGRITN	VHYAHKFANA	13.70%
TtAgo	-*Thermus thermophilus*	AVGFDAGGRE	AQAGERIPQE	LLLRDGRVPQ	LHLADRLVKE	20.17%
NgAgo	-*Natronobacterium gregoryi*	FIGIDVSRSY	PQLGEKLQST	VIHRDGFMNE	TAYADQASTH	11.41%
KmAgo	-*Kurthia massiliensis*	FIGIDVSHEN	ILAGEKIDDT	TIHRDGFWRE	IHYADLSATA	25.00%
DmAgo3	-*Drosophila melanogaster*	ICGIDSYHDP	TKREEIVNGL	IIYRDGIGDG	CMYAHKLAYL	30.74%
OsAgo7	-*Oryza sativa*	FMGADVTHPH	THRKEIIEQL	IFFRDGVSET	AYYAHLAAYR	27.94%
TcAgo	-*Tribolium castaneum*	FLGADVTHPP	QHRQEIIQEL	ILYRDGVSEG	AYYAHLVAFR	26.56%
BmAgo	-*Bombyx mori*	FLGVDVTHPP	QHRQEIVHEM	IMYRDGISEG	AYYAHLVAFR	27.23%
Ceagl1	-*Caenorhabditis elegans*	FFGCDITHPP	QHRQEIISDL	VVYRDGVSEG	AYYAHLVAFR	29.20%
QDE2	-*Neurospora crassa*	VVGYDVTHPT	PHGQESMTEQ	LIFRDGVSEG	AYYADLVCDR	46.71%
MusAgo2	-*Mus musculus*	FLGADVTHPP	QHRQEIIQDL	IFYRDGVSEG	AYYAHLVAFR	31.97%
PmAgo1	-*Penaeus monodon*	FLGADVTHPP	RQHDEVIQEL	ILYRDGVSEG	AYYAHLVAFR	26.75%
Amaub	-*Apis mellifera Aubergine*	VVGYDVCHDP	TTGEELSDEF	VIYRDGVGEG	CQYAHKLAFL	22.55%
Aaaub	-*Aedes aegypti Aubergine*	VIGFDVCHDT	TSGEELSNFM	IVYRDGVGEG	CQYAHKLAFL	24.46%
TtpAgo	-*Thermochaetoides thermophila*	FVGYDVTHPT	PKGQEQVDKV	IIFRDGVSEG	AYYADLVCTR	100.0%
TtpAgo QM	-*Thermochaetoides thermophila*	FVGYAVTHPT	PKGQAQVDKV	IIFRAGVSEG	AYYAALVCTR	

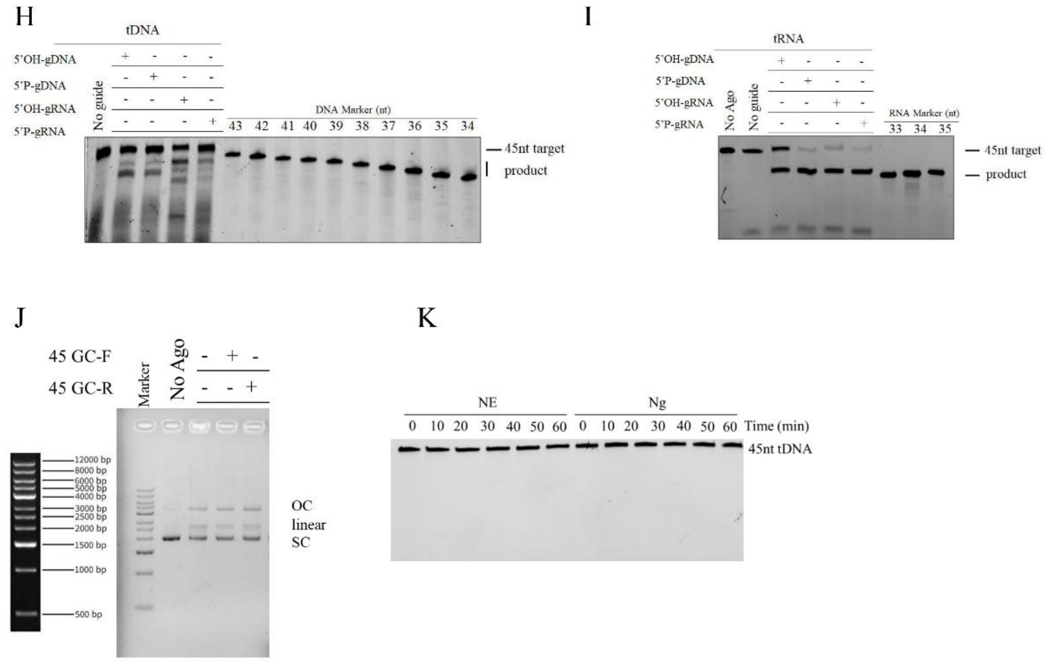

Supplementary Figure S1. Purification of TtpAgo protein, bioinformatics analysis, and *in vitro* biochemical activity analysis. (A) Maximum likelihood phylogenetic tree analysis of TtpAgo based on amino acid sequences. The numbers at the nodes indicate the bootstrap values for maximum likelihood analysis of 1000 resampled data sets. (B) The purity of the purified TtpAgo was determined using SDS-PAGE. (C) The purity of the purified TtpAgo-QM was determined using SDS-PAGE. (D) Nucleic acids associated with TtpAgo after heparin affinity and size-exclusion chromatography purification. Samples were treated with DNase I (D), RNase A (R), both nucleases (DR), or left untreated (-). (E, F) Nucleic acids associated with TtpAgo (E) or sfGFP (F) after Ni-NTA purification. Samples were treated with DNase I (D), RNase A (R), both nucleases (DR), or left untreated (-). (G) Multiple sequence alignment of a part of the PIWI domain from the TtpAgo with several other characterized Ago proteins. (H) Determining the DNA cleavage site with non-labeled tDNA. DNA marker (34, 35, 36, 37, 38, 39, 40, 41, 42, 43 nt) were partially hydrolyzed tDNA. (I) Determining the RNA cleavage site with FAM-labeled tRNA. RNA marker (33, 34, 35 nt) were chemically synthesized 5'-end, FAM-labeled. (J) Cleavage assays of double-stranded nucleic acids. (K) Time-course analysis of ssDNA degradation. NE, no enzyme; Ng, no guide.

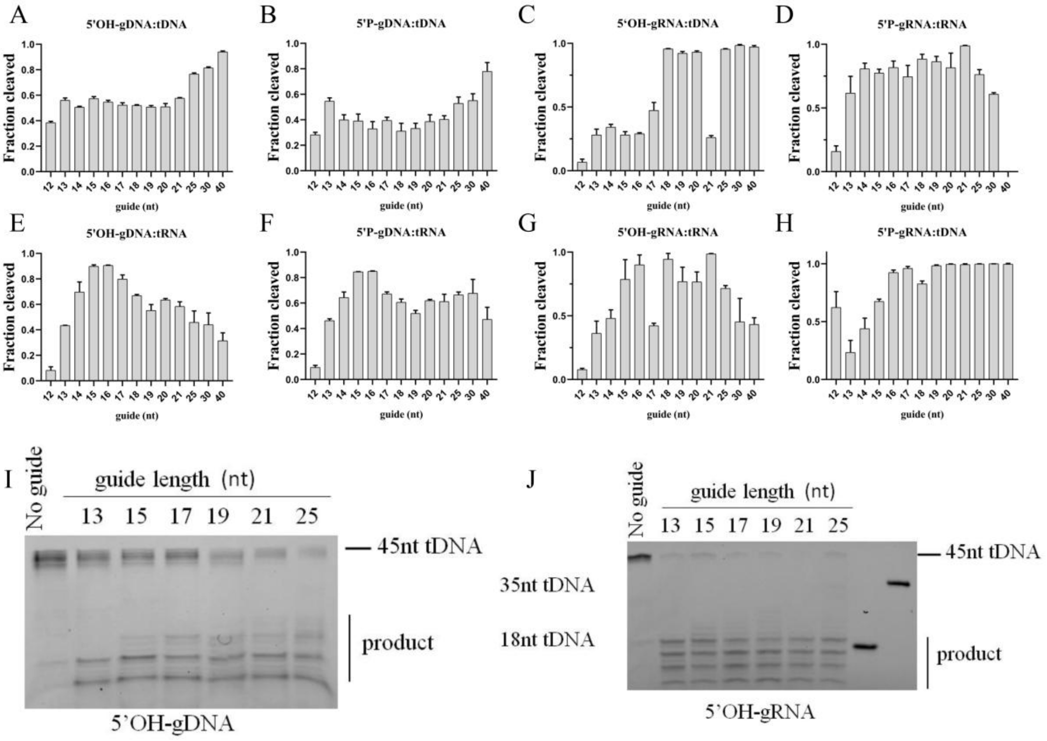

Supplementary Figure S2. Effects of the guide length on TtpAgo activity. (A) Effects of the 5’OH-gDNA length on DNA cleavage activity. (B) Effects of the 5’P-gDNA length on DNA cleavage activity. (C) Effects of the 5’OH-gRNA length on DNA cleavage activity. (D) Effects of the 5’P-gRNA length on RNA cleavage activity. (E) Effects of the 5’OH-gDNA length on RNA cleavage activity. (F) Effects of the 5’P-gDNA length on RNA cleavage activity. (G) Effects of the 5’OH-gRNA length on RNA cleavage activity. (H) Effects of the 5’P-gRNA length on DNA cleavage activity. Error bars represent SD of three independent experiments. Error bars represent SD of three independent experiments. (I) Guide DNA with 3’ end anchoring and 5’ end extension cleaves tDNA. (J) Guide RNA with 3’ end anchoring and 5’ end extension cleaves tDNA.

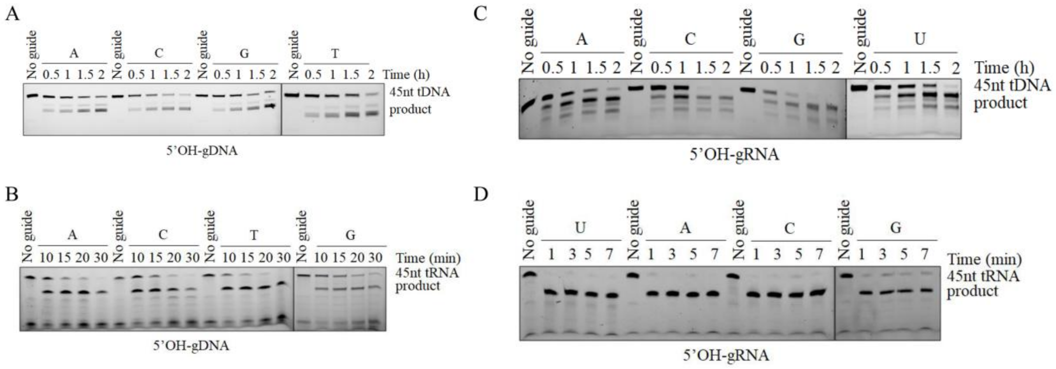

Supplementary Figure S3. Effects of the 5'-end nucleotide identity on TtpAgo activity. (A) Effects of the 5'-end nucleotide of 5'OH-gDNA on DNA cleavage activity. (B) Effects of the 5'-end nucleotide of 5'OH-gDNA on RNA cleavage activity. (C) Effects of the 5'-end nucleotide of 5'OH-gRNA on DNA cleavage activity. (D) Effects of the 5'-end nucleotide of 5'OH-gRNA on RNA cleavage activity. All experiments were performed at the 4:1:1 TtAgo:guide:target molar ratio in reaction buffer containing Mg^{2+} ions.

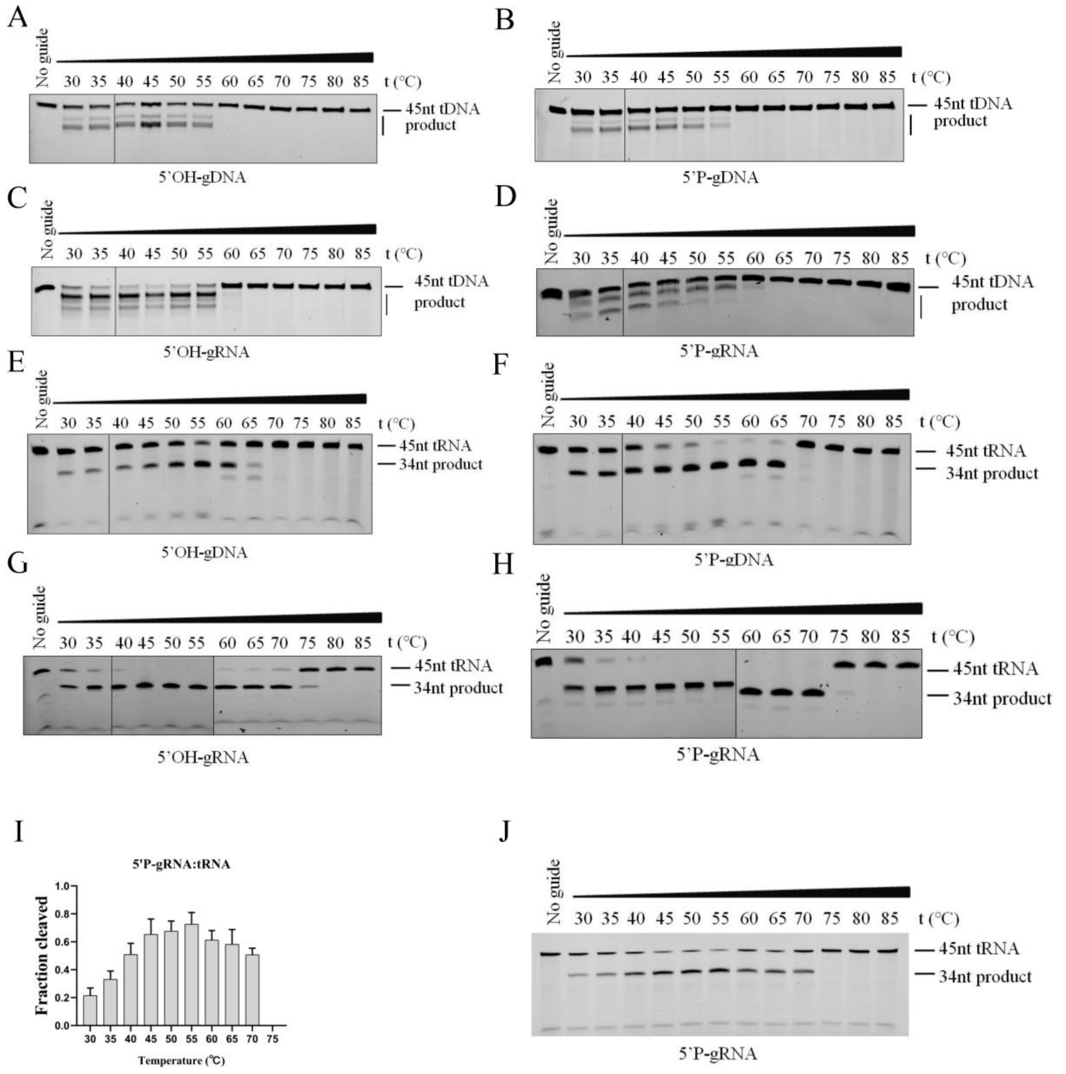

Supplementary Figure S4. Effects of temperature on TtpAgo activity. (A) Effects of temperature on DNA cleavage activity mediated by 5'OH-gDNA. (B) Effects of temperature on DNA cleavage activity mediated by 5'P-gDNA. (C) Effects of temperature on DNA activity mediated by 5'OH-gRNA. (D) Effects of temperature on DNA activity mediated by and 5'P-gRNA. (E) Effects of temperature on RNA activity mediated by 5'OH-gDNA. (F) Effects of temperature on RNA activity mediated by 5'P-gDNA. (G) Effects of temperature on RNA activity mediated by 5'OH-gRNA. (H) Effects of temperature on RNA activity mediated by 5'P-gRNA. (I) Effects of temperature on RNA activity mediated by 5'P-gRNA under the condition of halving the TtpAgo concentration. Error bars represent SD of three independent experiments. (J) Gel image of the effect of temperature on 5'P-gRNA-mediated RNA activity under the condition of halving the TtpAgo concentration.

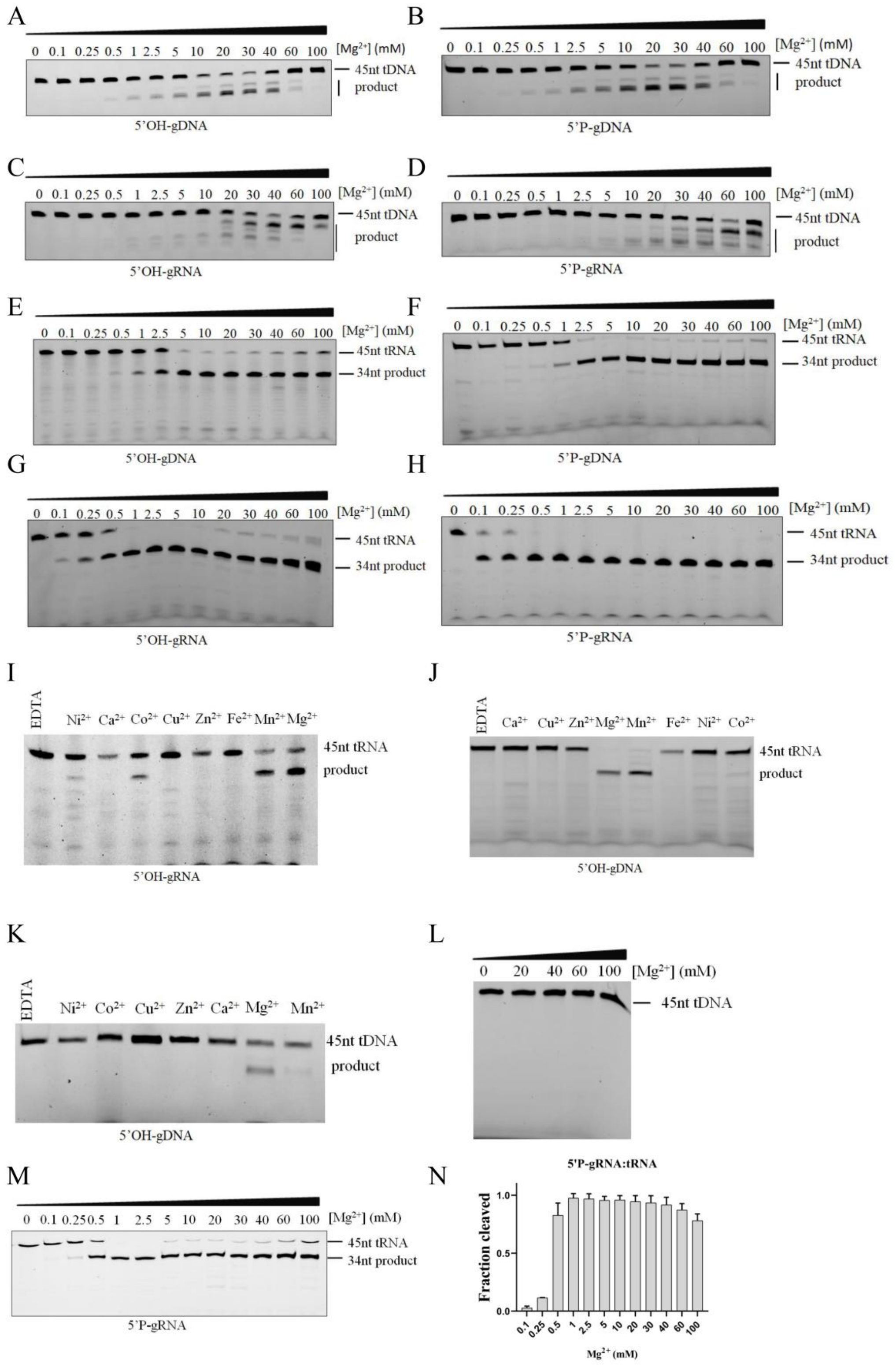

Supplementary Figure S5. Effects of Mg^{2+} concentration on TtpAgo cleavage activity. (A) Effects of Mg^{2+} ions concentration on DNA cleavage activity mediated by 5'OH-gDNA. (B) Effects of Mg^{2+} ions concentration on DNA cleavage activity mediated by 5'P-gDNA. (C) Effects of Mg^{2+} ions concentration on DNA cleavage activity mediated by 5'OH-gRNA. (D)

Effects of Mg^{2+} ions concentration on DNA cleavage activity mediated by 5'P-gRNA. (E) Effects of Mg^{2+} ions concentration on RNA cleavage activity mediated by 5'OH-gDNA. (F) Effects of Mg^{2+} ions concentration on RNA cleavage activity mediated by 5'P-gDNA. (G) Effects of Mg^{2+} ions concentration on RNA cleavage activity mediated by 5'OH-gRNA. (H) Effects of Mg^{2+} ions concentration on RNA cleavage activity mediated by 5'P-gRNA. (I) Effects of different divalent metal ions on RNA cleavage activity mediated by 5'OH-gRNA. (J) Effects of different divalent metal ions on RNA cleavage activity mediated by 5'OH-gDNA. (K) Effects of different divalent metal ions on DNA cleavage activity mediated by 5'OH-gDNA. (L) Effects of different concentrations of magnesium ions on single-stranded DNA. (M) Gel image of the effect of Mg^{2+} ions concentration on 5'P-gRNA-mediated RNA activity under the condition of halving the TtpAgo concentration. (N) Effects of Mg^{2+} ions concentration on RNA activity mediated by 5'P-gRNA under the condition of halving the TtpAgo concentration. Error bars represent SD of three independent experiments. The assay in (A) was performed in 45 °C for 120 min at indicated Mg^{2+} ions concentration. The assay in (B) was performed in 40 °C for 120 min at indicated Mg^{2+} ions concentration. The assay in (C) was performed in 45 °C for 120 min at indicated Mg^{2+} ions concentration. The assay in (D) was performed in 40 °C for 120 min at indicated Mg^{2+} ions concentration. The assay in (E), 5'OH-gDNA:RNA was performed in 55 °C for 30 min at indicated Mg^{2+} ions concentration. The assay in (F), 5'P-gDNA:RNA was performed in 55 °C for 20 min at indicated Mg^{2+} ions concentration. The assay in (G), (H) were performed in 55 °C for 3 min at indicated Mg^{2+} ions concentration.

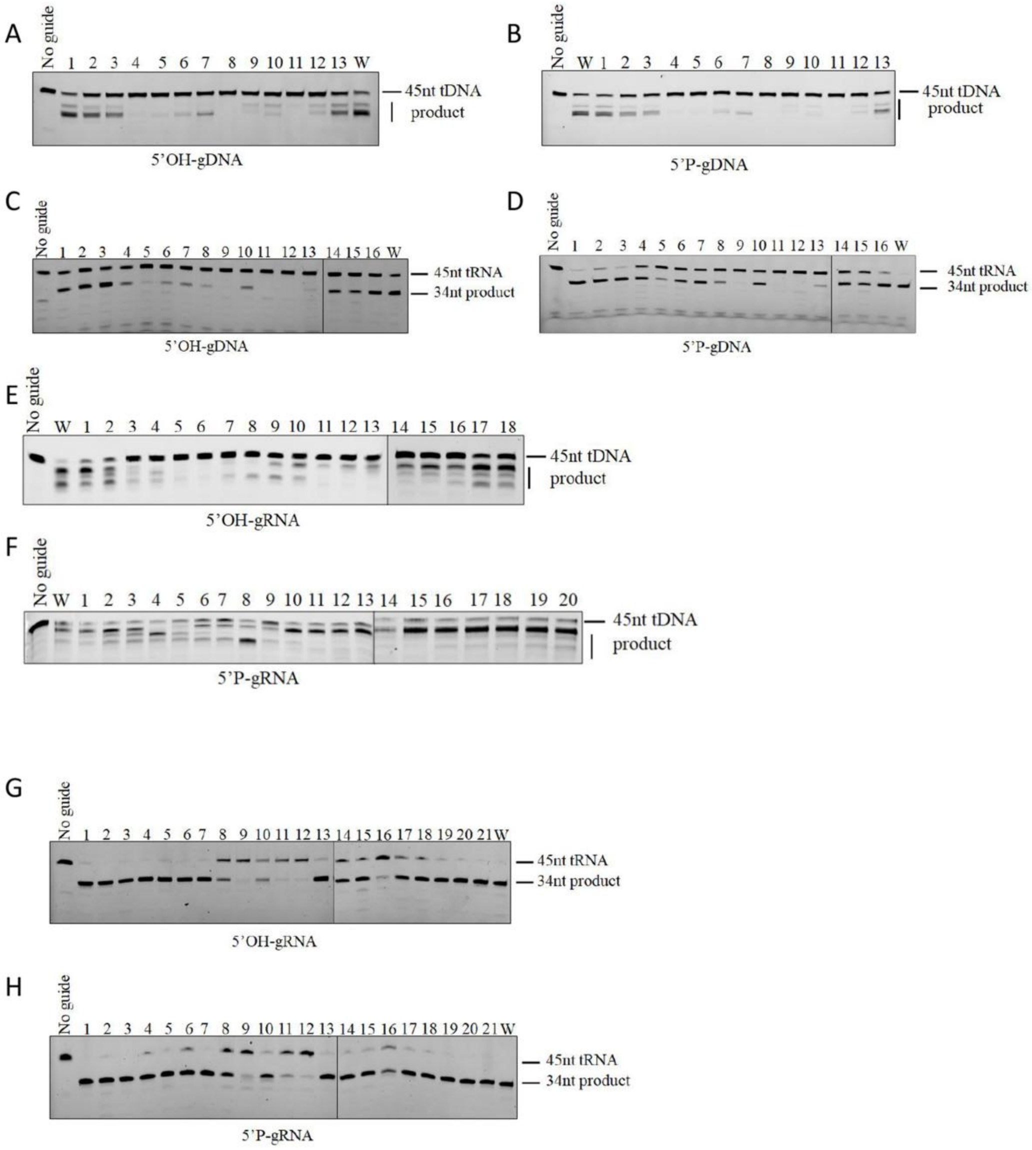

Supplementary Figure S6. Effects of mismatch position on TtpAgo activity.

(A) 5’OH-gDNA:tDNA was performed at the 4:1:1 TtpAgo:guide:target molar ratio in reaction buffer containing Mg^{2+} ions for 120 min at 45°C. (B) 5’P-gDNA:tDNA was performed at the 4:1:1 TtpAgo:guide:target molar ratio in reaction buffer containing Mg^{2+} ions for 120 min at 40°C. (C) 5’OH-gDNA:tRNA were performed at the 4:1:1 TtpAgo:guide:target molar ratio in reaction buffer containing Mg^{2+} ions for 30 min at 55°C. (D) 5’P-gDNA :tRNA was performed at the 4:1:1 TtpAgo:guide:target molar ratio in reaction buffer containing Mg^{2+} ions for 20min at 55°C. (E) 5’OH-gRNA :tDNA was performed at the 4:1:1 TtpAgo:guide:target molar ratio in reaction buffer containing Mg^{2+} ions for 120min at 45°C . (F) 5’P-gRNA :tDNA were performed at the 4:1:1 TtpAgo:guide:target molar ratio in

reaction buffer containing Mg^{2+} ions for 120 min at 40°C. (G) and (H) 5’OH-gRNA:tRNA and 5’P-gRNA:tRNA were performed at the 4:1:1 TtpAgo:guide:target molar ratio in reaction buffer containing Mg^{2+} ions for 1 min at 55°C. Error bars represent SD of three independent experiments. W, no mismatch.

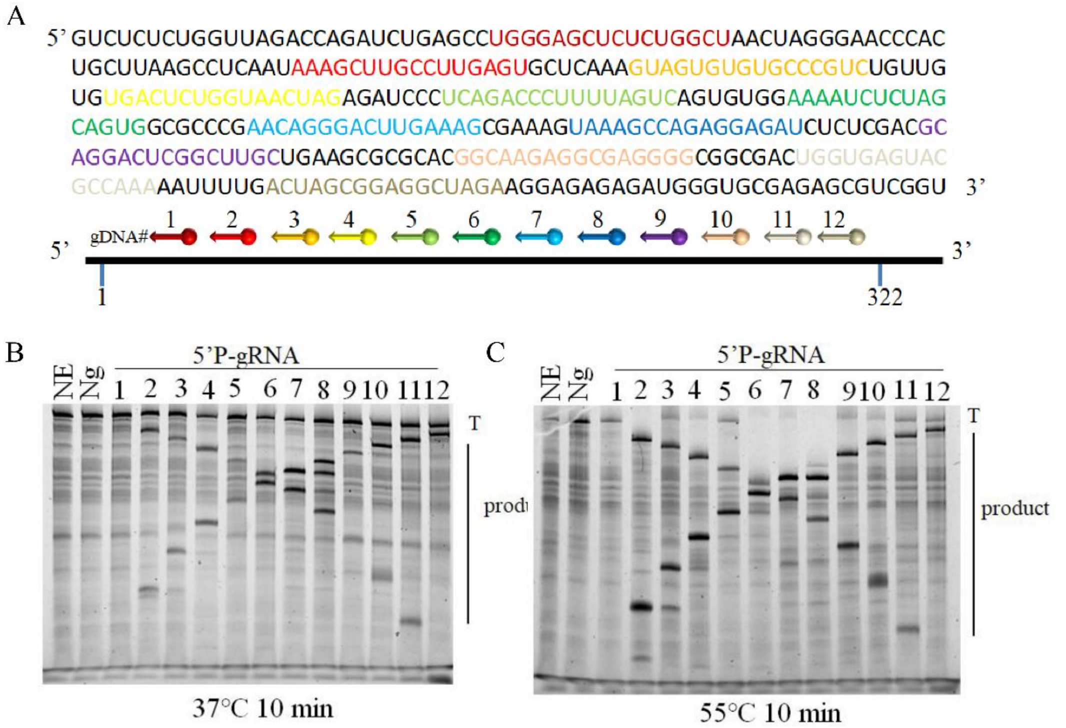

Supplementary Figure S7. Cleavage activity of TtpAgo complex for highly RNA structures. (A) Schematic overview of the HIV-1 ΔDIS 5'UTR. Arrows with different colors indicate the target region and the corresponding gDNAs and gRNAS are numbered from 1 to 12 with the corresponding colours. (B) Substrates and products generated by the assay described in Figure 7A were resolved by denaturing PAGE (8%) revealing cleavability of the highly structured RNA by TtpAgo–gRNA complex in 37℃. (C) Substrates and products generated by the assay described in Figure 7A were resolved by denaturing PAGE (8%) revealing cleavability of the highly structured RNA by TtpAgo–gRNA complex in 55℃. T, Target RNA; NE, no enzyme; Ng, no guide.

A MID-domain

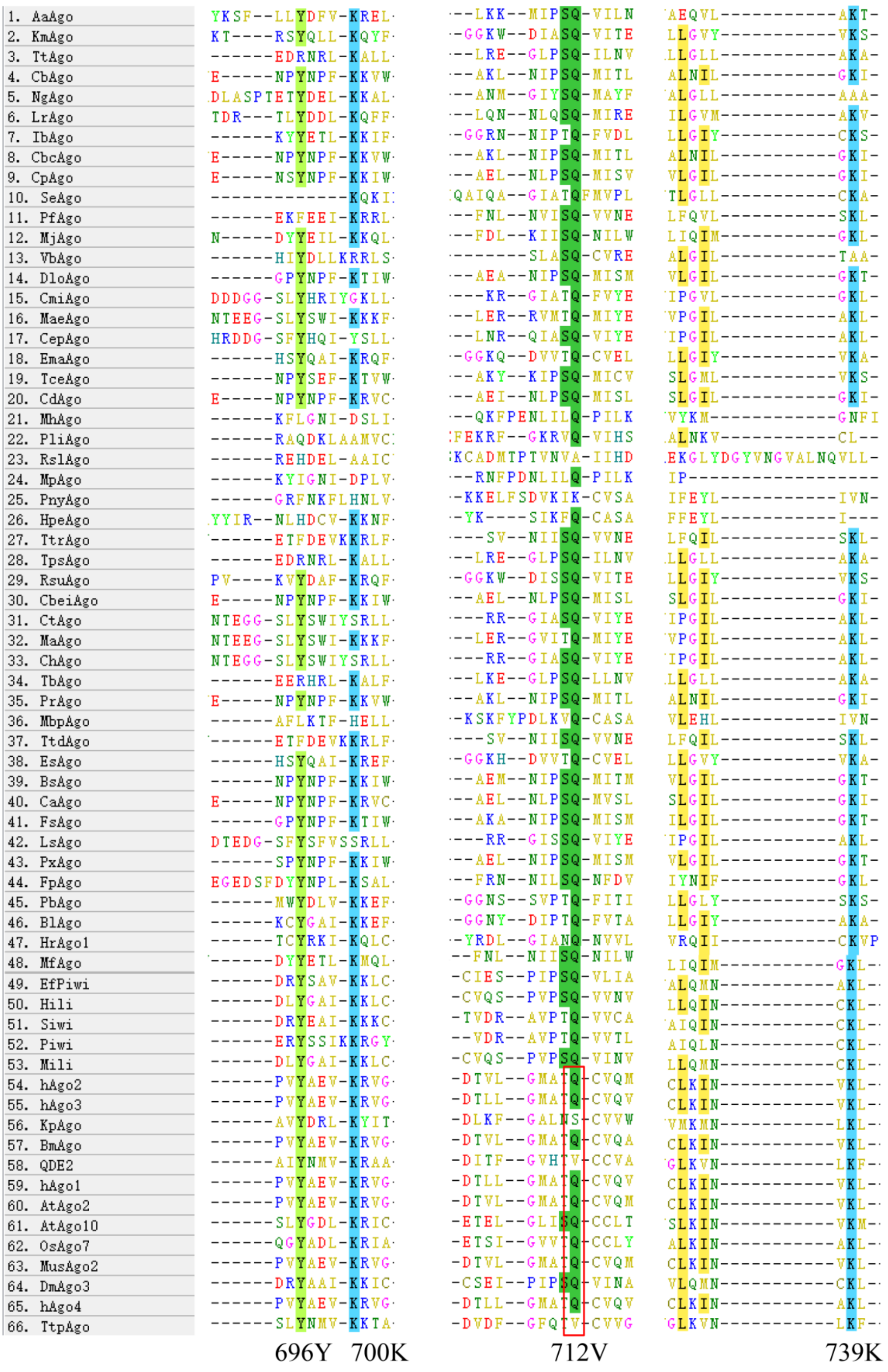
1. AaAgo
2. KmAgo
3. TtAgo
4. CbAgo
5. NgAgo
6. LrAgo
7. IbAgo
8. CbcAgo
9. CpAgo
10. SeAgo
11. PfAgo
12. MjAgo
13. VbAgo
14. DloAgo
15. CmiAgo
16. MaeAgo
17. CepAgo
18. EmaAgo
19. TceAgo
20. CdAgo
21. MhAgo
22. PliAgo
23. RslAgo
24. MpAgo
25. PnyAgo
26. HpeAgo
27. TtrAgo
28. TpsAgo
29. RsuAgo
30. CbeiAgo
31. CtAgo
32. MaAgo
33. ChAgo
34. TbAgo
35. PrAgo
36. MbpAgo
37. TtdAgo
38. EsAgo
39. BsAgo
40. CaAgo
41. FsAgo
42. LsAgo
43. PxAgo
44. FpAgo
45. PbAgo
46. BlAgo
47. HrAgo1
48. MfAgo
49. EfPiwi
50. Hili
51. Siwi
52. Piwi
53. Mili
54. hAgo2
55. hAgo3
56. KpAgo
57. BmAgo
58. QDE2
59. hAgo1
60. AtAgo2
61. AtAgo10
62. OsAgo7
63. MusAgo2
64. DmAgo3
65. hAgo4
66. TtpAgo
696Y
700K
712V
739K

B PIWI-domain

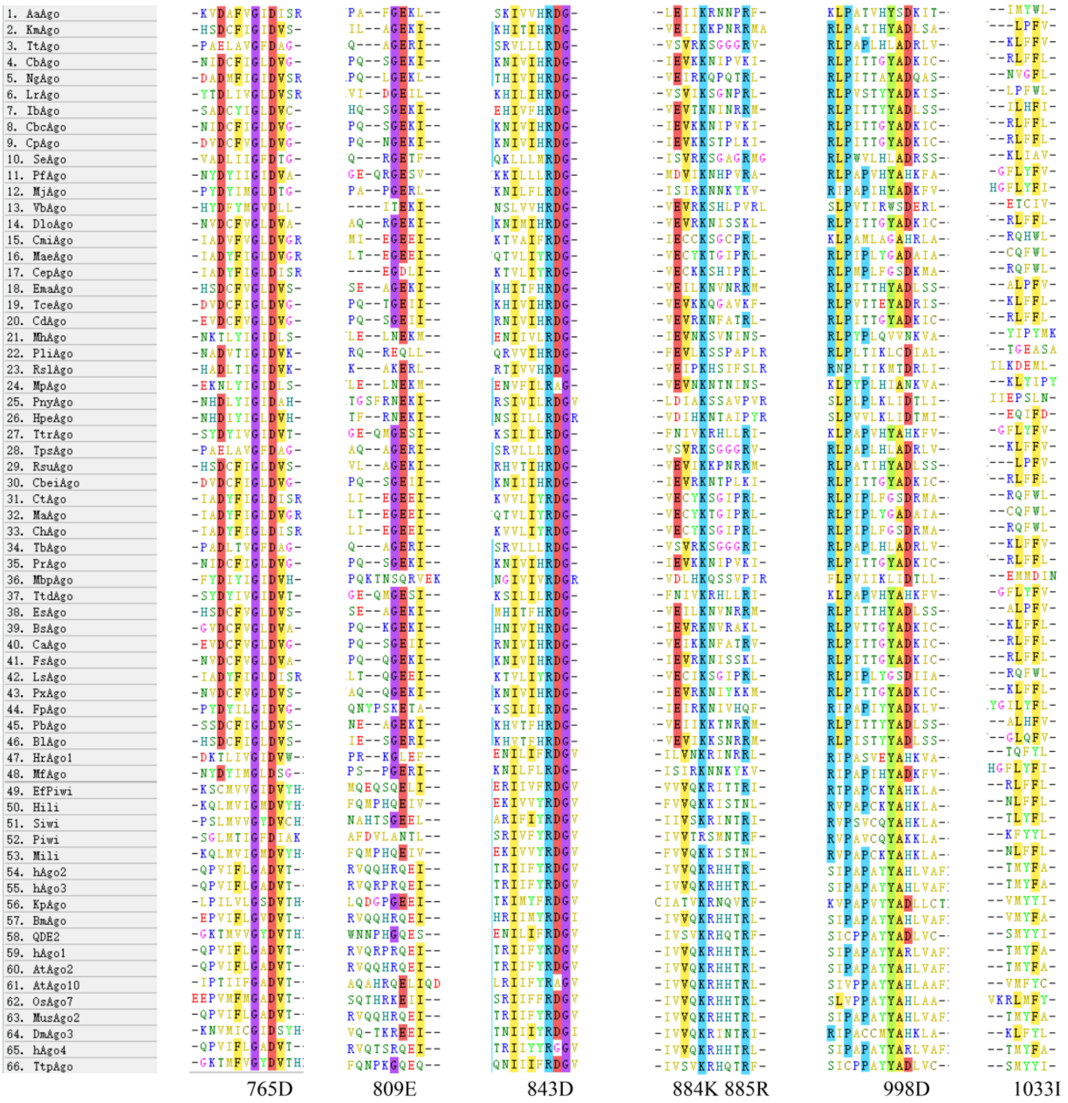

C PAZ-domain

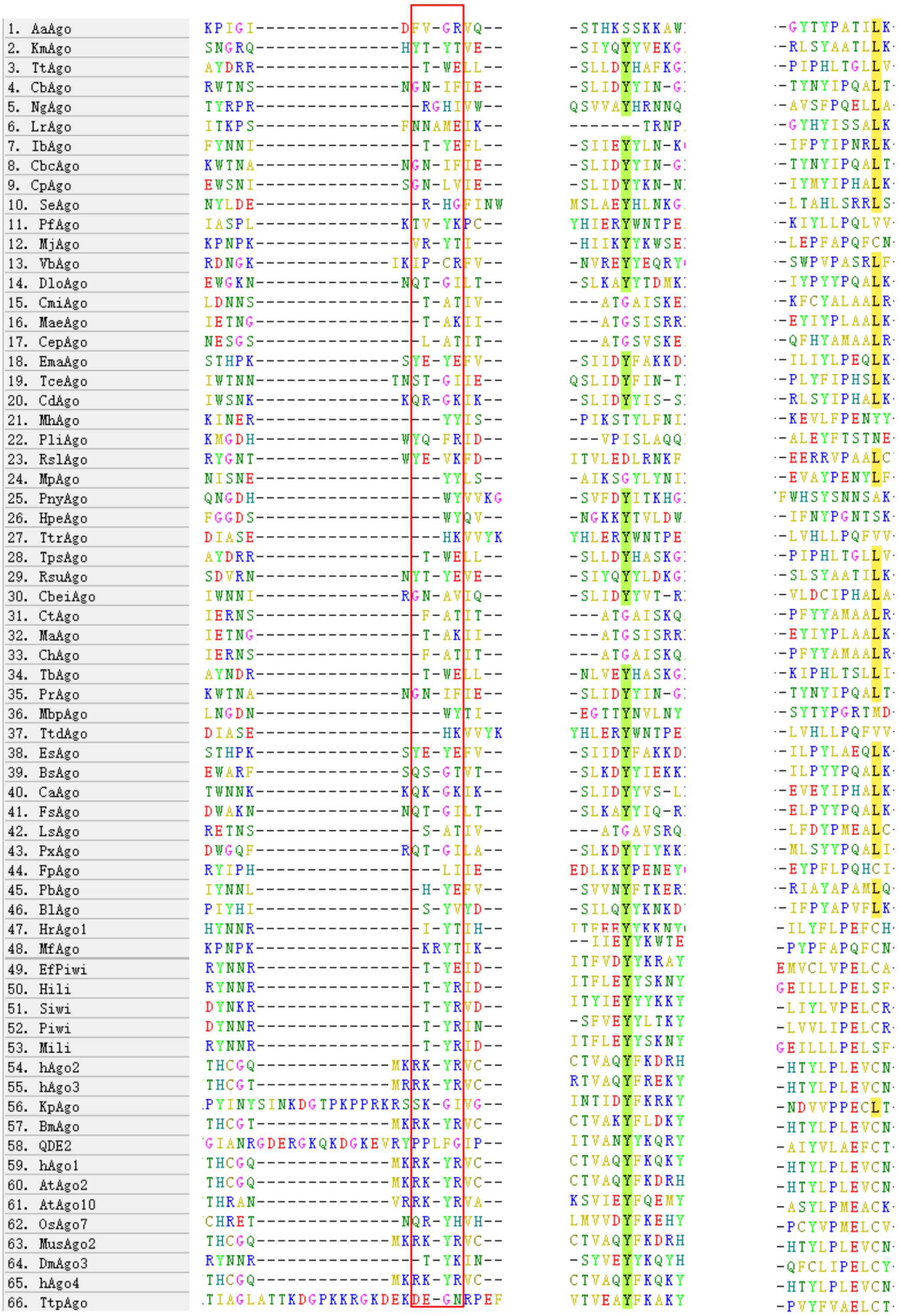

430D 432G 477A 478Y 505F

Supplementary Figure S8. Multiple sequence alignment of TtpAgo with the characterized Agos. (A) Sequence alignment of some MID domains. (B) Sequence alignment of some PIWI domains. (C) Sequence alignment of some PAZ domains.

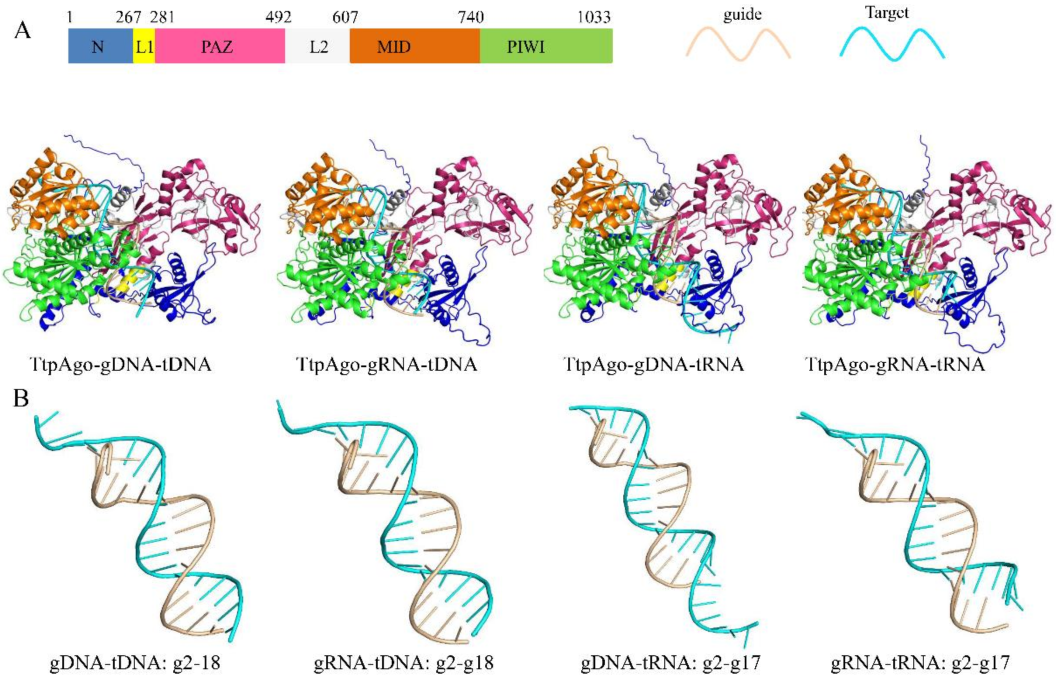

Supplementary Figure S9. TtpAgo structure analysis. (A) (Upper) Schematic representation of TtpAgo domains: N-terminal (blue), L1 (yellow), PAZ (pink), L2 (gray), MID (orange), and PIWI (green). (Lower panal) Structural models of TtpAgo with different guides and targets. (B) Close-up view showing only the guide and target strands.

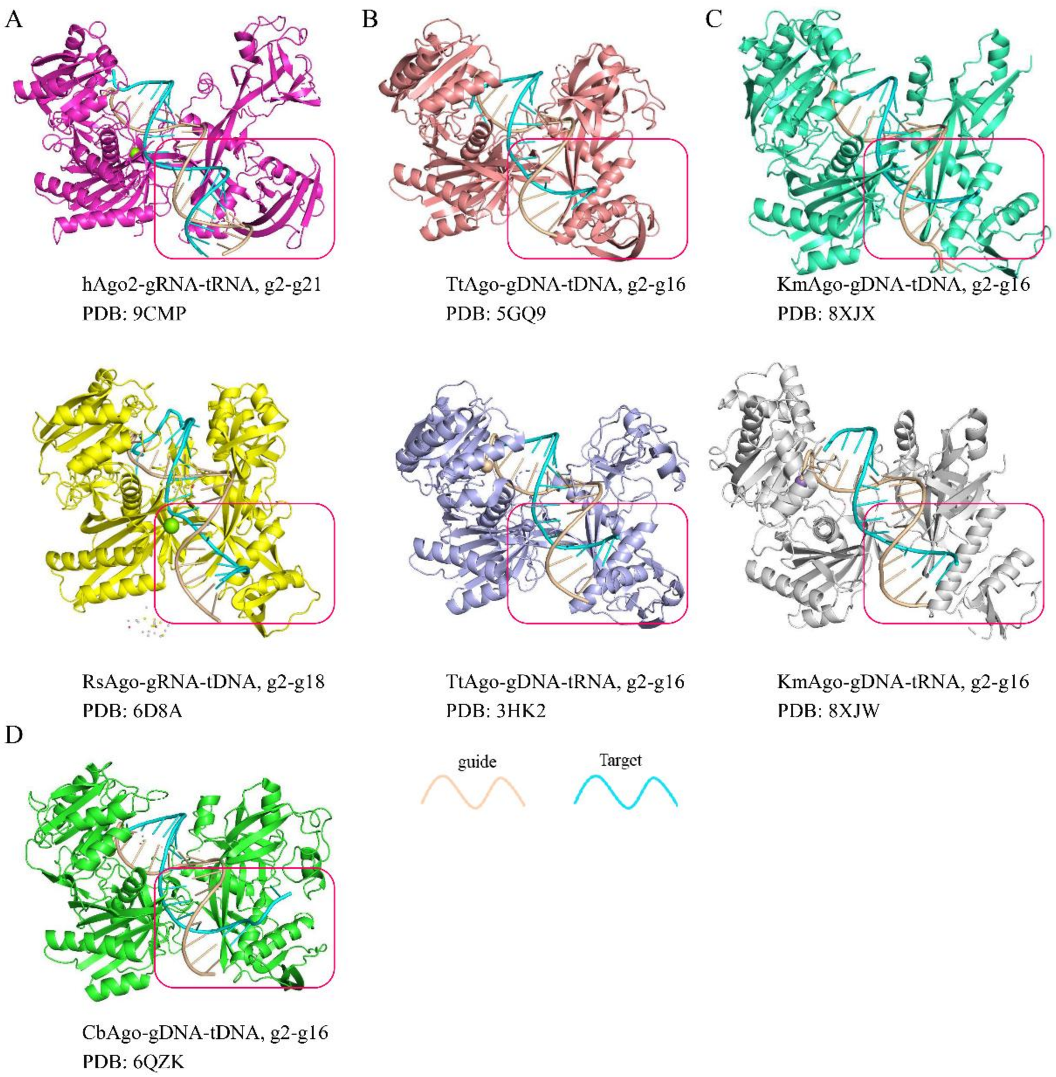

Supplementary Figure S10. Ago structures. (A) Target recognition model of the packing-type N-terminal domain in hAgo2 and RsAgo. (B) Target recognition model of the wedge-type N-terminal domain in TtAgo. (C) Target recognition model of the wedge-type N-terminal domain in KmAgo. (D) Target recognition model of the wedge-type N-terminal domain in CbAgo.

Supplementary Table S1. List of sequence of gDNAs and gRNAs targeting DNA or RNA

Oligonucleotide name	Sequence (5’-3’)	Description
FAM-T-tDNA	FAM-AAACGACGGCCAGTGCCAAGCT TACTATACAACCT ACTACCTCAT	5’ FAM labeled T-tDNA
FAM-U-tRNA	FAM-AAACGACGGCCAGUGCCAAGC UUACUAUACAACC UACUACCUCAU	5’ FAM labeled T-tRNA
16 nt T-gDNA	TGAGGTAGTAGGTTGT	16 nt guide forms 5’-T pair with T-tRNA
16 nt A-gDNA	AGAGGTAGTAGGTTGT	16 nt guide forms 5’-A pair with A-tRNA
16 nt C-gDNA	CGAGGTAGTAGGTTGT	16 nt guide forms 5’-C pair with C-tRNA
16 nt G-gDNA	GGAGGTAGTAGGTTGT	16 nt guide forms 5’-G pair with G-tRNA
13 nt T-gDNA	TGAGGTAGTAGGT	13 nt guide forms 5’-T pair with T-tDNA
13 nt A-gDNA	AGAGGTAGTAGGT	13 nt guide forms 5’-A pair with A-tDNA
13 nt C-gDNA	CGAGGTAGTAGGT	13 nt guide forms 5’-C pair with C-tDNA
13 nt G-gDNA	GGAGGTAGTAGGT	13 nt guide forms 5’-G pair with G-tDNA
18 nt U-gRNA	UGAGGUAGUAGGUUGUAU	18 nt guide forms 5’-U pair with T-tDNA
18 nt A-gRNA	AGAGGUAGUAGGUUGUAU	18 nt guide forms 5’-A pair with A-tDNA
18 nt C-gRNA	CGAGGUAGUAGGUUGUAU	18 nt guide forms 5’-C pair with C-tDNA

18 nt G-gRNA	GGAGGUAGUAGGUUGUAU	18 nt guide forms 5’-G pair with G-tDNA
20 nt U-gRNA	UGAGGUAGUAGGUUGUAUAG	20 nt guide forms 5’-U pair with T-tDNA
20 nt A-gRNA	AGAGGUAGUAGGUUGUAUAG	20 nt guide forms 5’-U pair with A-tDNA
20 nt C-gRNA	CGAGGUAGUAGGUUGUAUAG	20 nt guide forms 5’-U pair with C-tDNA
20 nt G-gRNA	GGAGGUAGUAGGUUGUAUAG	20 nt guide forms 5’-U pair with G-tDNA
21 nt U-gRNA	UGAGGUAGUAGGUUGUAUAGU	21 nt guide forms 5’-U pair with T-tRNA
21 nt A-gRNA	AGAGGUAGUAGGUUGUAUAGU	21 nt guide forms 5’-A pair with A-tRNA
21 nt C-gRNA	CGAGGUAGUAGGUUGUAUAGU	21 nt guide forms 5’-C pair with C-tRNA
21 nt G-gRNA	GGAGGUAGUAGGUUGUAUAGU	21 nt guide forms 5’-G pair with G-tRNA
33 nt DNA product	AAACGACGGCCAGTGCC AAGCTTACTATACAAC	33 nt DNA marker
34 nt DNA product	AAACGACGGCCAGTGCC AAGCTTACTATACAACC	34 nt DNA marker
35 nt DNA product	AAACGACGGCCAGTGCC AAGCTTACTATACAACCT	35 nt DNA marker
36 nt DNA product	AAACGACGGCCAGTGCC AAGCTTACTATACAACCT A	36 nt DNA marker
37 nt DNA product	AAACGACGGCCAGTGCC AAGCTTACTATACAACCT AC	37 nt DNA marker
38 nt DNA product	AAACGACGGCCAGTGCC	38 nt DNA marker

	AAGCTTACTATACAACCT ACT	
39 nt DNA product	AAACGACGGCCAGTGCC AAGCTTACTATACAACCT ACTA	39 nt DNA marker
40 nt DNA product	AAACGACGGCCAGTGCC AAGCTTACTATACAACCT ACTAC	40 nt DNA marker
41 nt DNA product	AAACGACGGCCAGTGCC AAGCTTACTATACAACCT ACTACC	41 nt DNA marker
33 nt RNA product	FAM-AAACGACGGCCAGUGCC AAGCUUACUAUACAAC	33 nt RNA marker
34 nt RNA product	FAM-AAACGACGGCCAGUGCC AAGCUUACUAUACAACC	34 nt RNA marker
35 nt RNA product	FAM-AAACGACGGCCAGUGCC AAGCUUACUAUACAACC U	35 nt RNA marker
FAM-A-tDNA	FAM-AAACGACGGCCAGTGCCAAGCT T ACTATACAACCTACTACCTCTT	let-7 based 45 nt DNA target for A-gDNA/A-gRNA
FAM-G-tDNA	FAM-AAACGACGGCCAGTGCCAAGCT T ACTATACAACCTACTACCTCGT	let-7 based 45 nt DNA target for G-gDNA/G-gRNA
T-tDNA	FAM-AAACGACGGCCAGTGCCAAGCT T ACTATACAACCTACTACCTCAT	let-7 based 45 nt DNA target for T-gDNA/U-gRNA
FAM-C-tDNA	FAM-AAACGACGGCCAGTGCCAAGCT T ACTATACAACCTACTACCTCGT	let-7 based 45 nt DNA target for C-gDNA/C-gRNA
FAM-C-tRNA	FAM-AAACGACGGCCAGUGCCAAGC UU ACUAUACAACCUACUACCUCGU	let-7 based 45 nt RNA target for C-gRNA/C-gDNA
FAM-U-tRNA	FAM-AAACGACGGCCAGUGCCAAGC UU ACUAUACAACCUACUACCUCAU	let-7 based 45 nt RNA target for U-gDNA/U-gRNA
FAM-A-tRNA	FAM-AAACGACGGCCAGUGCCAAGC UU ACUAUACAACCUACUACCUCUU	let-7 based 45 nt RNA target for A-gDNA/A-gRNA
FAM-G-tRNA	FAM-AAACGACGGCCAGUGCCAAGC UU ACUAUACAACCUACUACCUCCU	let-7 based 45 nt RNA target for G-gDNA/G-gRNA

13 nt gDNA_mm1	AGAGGTAGTAGGT	guide forms mismatched pair in position 1 with T-tDNA
13 nt gDNA_mm2	TCAGGTAGTAGGT	guide forms mismatched pair in position 2 with T-tDNA
13 nt gDNA_mm3	TGTGGTAGTAGGT	guide forms mismatched pair in position 3 with T-tDNA
13 nt gDNA_mm4	TGACGTAGTAGGT	guide forms mismatched pair in position 4 with T-tDNA
13 nt gDNA_mm5	TGAGCTAGTAGGT	guide forms mismatched pair in position 5 with T-tDNA
13 nt gDNA_mm6	TGAGGAAGTAGGT	guide forms mismatched pair in position 6 with T-tDNA
13 nt gDNA_mm7	TGAGGTTGTAGGT	guide forms mismatched pair in position 7 with T-tDNA
13 nt gDNA_mm8	TGAGGTACTAGGT	guide forms mismatched pair in position 8 with T-tDNA
13 nt gDNA_mm9	TGAGGTAGAAGGT	guide forms mismatched pair in position 9 with T-tDNA
13 nt gDNA_mm10	TGAGGTAGTTGGT	guide forms mismatched pair in position 10 with T-tDNA
13 nt gDNA_mm11	TGAGGTAGTACGT	guide forms mismatched pair in position 11 with T-tDNA
13 nt gDNA_mm12	TGAGGTAGTAGCT	guide forms mismatched pair in position 12 with T-tDNA
13 nt gDNA_mm13	TGAGGTAGTAGGA	guide forms mismatched pair in position 13 with T-tDNA
16 nt gDNA_mm1	AGAGGTAGTAGGTTGT	guide forms mismatched pair in position 1 with T-tRNA
16 nt gDNA_mm2	ACAGGTAGTAGGTTGT	guide forms mismatched pair in

		position 2 with T-tRNA
16 nt gDNA_mm3	AGTGGTAGTAGGTTGT	guide forms mismatched pair in position 3 with T-tRNA
16 nt gDNA_mm4	AGACGTAGTAGGTTGT	guide forms mismatched pair in position 4 with T-tRNA
16 nt gDNA_mm5	AGAGCTAGTAGGTTGT	guide forms mismatched pair in position 5 with T-tRNA
16 nt gDNA_mm6	AGAGGAAGTAGGTTGT	guide forms mismatched pair in position 6 with T-tRNA
16 nt gDNA_mm7	AGAGGTTGTAGGTTGT	guide forms mismatched pair in position 7 with T-tRNA
16 nt gDNA_mm8	AGAGGTACTAGGTTGT	guide forms mismatched pair in position 8 with T-tRNA
16 nt gDNA_mm9	AGAGGTAGAAGGTTGT	guide forms mismatched pair in position 9 with T-tRNA
16 nt gDNA_mm10	AGAGGTAGTTGGTTGT	guide forms mismatched pair in position 10 with T-tRNA
16 nt gDNA_mm11	AGAGGTAGTACGTTGT	guide forms mismatched pair in position 11 with T-tRNA
16 nt gDNA_mm12	AGAGGTAGTAGCTTGT	guide forms mismatched pair in position 12 with T-tRNA
16 nt gDNA_mm13	AGAGGTAGTAGGATGT	guide forms mismatched pair in position 13 with T-tRNA
16 nt gDNA_mm14	AGAGGTAGTAGGTAGT	guide forms mismatched pair in position 14 with T-tRNA
16 nt gDNA_mm15	AGAGGTAGTAGGTTCT	guide forms mismatched pair in position 15 with T-tRNA
16 nt gDNA_mm16	AGAGGTAGTAGGTTGA	guide forms mismatched pair in position 16 with T-tRNA

18 nt gRNA_mm1	UGAGGUAGUAGGUUGUAU	guide forms mismatched pair in position 1 with T-tDNA
18 nt gRNA_mm2	UCAGGUAGUAGGUUGUAU	guide forms mismatched pair in position 2 with T-tDNA
18 nt gRNA_mm3	UGUGGUAGUAGGUUGUAU	guide forms mismatched pair in position 3 with T-tDNA
18 nt gRNA_mm4	UGACGUAGUAGGUUGUAU	guide forms mismatched pair in position 4 with T-tDNA
18 nt gRNA_mm5	UGAGCUAGUAGGUUGUAU	guide forms mismatched pair in position 5with T-tDNA
18 nt gRNA_mm6	UGAGGAAGUAGGUUGUAU	guide forms mismatched pair in position 6 with T-tDNA
18 nt gRNA_mm7	UGAGGUUGUAGGUUGUAU	guide forms mismatched pair in position 7 with T-tDNA
18 nt gRNA_mm8	UGAGGUACUAGGUUGUAU	guide forms mismatched pair in position 8 with T-tDNA
18 nt gRNA_mm9	UGAGGUAGAAGGUUGUAU	guide forms mismatched pair in position 9 with T-tDNA
18 nt gRNA_mm10	UGAGGUAGUUGGUUGUAU	guide forms mismatched pair in position 10 with T-tDNA
18 nt gRNA_mm11	UGAGGUAGUACGUUGUAU	guide forms mismatched pair in position 11 with T-tDNA
18 nt gRNA_mm12	UGAGGUAGUAGCUUGUAU	guide forms mismatched pair in position 12 with T-tDNA
18 nt gRNA_mm13	UGAGGUAGUAGGAUGUAU	guide forms mismatched pair in position 13 with T-tDNA
18 nt gRNA_mm14	UGAGGUAGUAGGUAGUAU	guide forms mismatched pair in position 14 with T-tDNA
18 nt gRNA_mm15	UGAGGUAGUAGGUUCUAU	guide forms mismatched pair in

		position 15 with T-tDNA
18 nt gRNA_mm16	UGAGGUAGUAGGUUGAAU	guide forms mismatched pair in position 16 with T-tDNA
18 nt gRNA_mm17	UGAGGUAGUAGGUUGUUU	guide forms mismatched pair in position 17 with T-tDNA
18 nt gRNA_mm18	UGAGGUAGUAGGUUGUAA	guide forms mismatched pair in position 18 with T-tDNA
20 nt gRNA_mm1	UGAGGUAGUAGGUUGUAUAG	guide forms mismatched pair in position 1 with T-tDNA
20 nt gRNA_mm2	UCAGGUAGUAGGUUGUAUAG	guide forms mismatched pair in position 2 with T-tDNA
20 nt gRNA_mm3	UGUGGUAGUAGGUUGUAUAG	guide forms mismatched pair in position 3 with T-tDNA
20 nt gRNA_mm4	UGACGUAGUAGGUUGUAUAG	guide forms mismatched pair in position 4 with T-tDNA
20 nt gRNA_mm5	UGAGCUAGUAGGUUGUAUAG	guide forms mismatched pair in position 5with T-tDNA
20 nt gRNA_mm6	UGAGGAAGUAGGUUGUAUAG	guide forms mismatched pair in position 6 with T-tDNA
20 nt gRNA_mm7	UGAGGUUGUAGGUUGUAUAG	guide forms mismatched pair in position 7 with T-tDNA
20 nt gRNA_mm8	UGAGGUACUAGGUUGUAUAG	guide forms mismatched pair in position 8 with T-tDNA
20 nt gRNA_mm9	UGAGGUAGAAGGUUGUAUAG	guide forms mismatched pair in position 9 with T-tDNA
20 nt gRNA_mm10	UGAGGUAGUUGGUUGUAUAG	guide forms mismatched pair in position 10 with T-tDNA
20 nt gRNA_mm11	UGAGGUAGUACGUUGUAUAG	guide forms mismatched pair in position 11 with T-tDNA

20 nt gRNA_mm12	UGAGGUAGUAGCUUGUAUAG	guide forms mismatched pair in position 12 with T-tDNA
20 nt gRNA_mm13	UGAGGUAGUAGGAUGUAUAG	guide forms mismatched pair in position 13 with T-tDNA
20 nt gRNA_mm14	UGAGGUAGUAGGUAGUAUAG	guide forms mismatched pair in position 14 with T-tDNA
20 nt gRNA_mm15	UGAGGUAGUAGGUUCUAUAG	guide forms mismatched pair in position 15 with T-tDNA
20 nt gRNA_mm16	UGAGGUAGUAGGUUGAAUAG	guide forms mismatched pair in position 16 with T-tDNA
20 nt gRNA_mm17	UGAGGUAGUAGGUUGUUUAG	guide forms mismatched pair in position 17 with T-tDNA
20 nt gRNA_mm18	UGAGGUAGUAGGUUGUAAAG	guide forms mismatched pair in position 18 with T-tDNA
20 nt gRNA_mm19	UGAGGUAGUAGGUUGUAUUG	guide forms mismatched pair in position 19 with T-tDNA
20 nt gRNA_mm20	UGAGGUAGUAGGUUGUAUAC	guide forms mismatched pair in position 20 with T-tDNA
21 nt gRNA_mm1	UGAGGUAGUAGGUUGUAUAGU	guide forms mismatched pair in position 1 with T-tRNA
21 nt gRNA_mm2	UCAGGUAGUAGGUUGUAUAGU	guide forms mismatched pair in position 2 with T-tRNA
21 nt gRNA_mm3	UGUGGUAGUAGGUUGUAUAGU	guide forms mismatched pair in position 3 with T-tRNA
21 nt gRNA_mm4	UGACGUAGUAGGUUGUAUAGU	guide forms mismatched pair in position 4 with T-tRNA
21 nt gRNA_mm5	UGAGCUAGUAGGUUGUAUAGU	guide forms mismatched pair in position 5with T-tRNA

21 nt gRNA_mm6	UGAGGAAGUAGGUUGUAUAGU	guide forms mismatched pair in position 6 with T-tRNA
21 nt gRNA_mm7	UGAGGUUGUAGGUUGUAUAGU	guide forms mismatched pair in position 7 with T-tRNA
21 nt gRNA_mm8	UGAGGUACUAGGUUGUAUAGU	guide forms mismatched pair in position 8 with T-tRNA
21 nt gRNA_mm9	UGAGGUAGAAGGUUGUAUAGU	guide forms mismatched pair in position 9 with T-tRNA
21 nt gRNA_mm10	UGAGGUAGUUGGUUGUAUAGU	guide forms mismatched pair in position 10 with T-tRNA
21 nt gRNA_mm11	UGAGGUAGUACGUUGUAUAGU	guide forms mismatched pair in position 11 with T-tRNA
21 nt gRNA_mm12	UGAGGUAGUAGCUUGUAUAGU	guide forms mismatched pair in position 12 with T-tRNA
21 nt gRNA_mm13	UGAGGUAGUAGGAUGUAUAGU	guide forms mismatched pair in position 13 with T-tRNA
21 nt gRNA_mm14	UGAGGUAGUAGGUAGUAUAGU	guide forms mismatched pair in position 14 with T-tRNA
21 nt gRNA_mm15	UGAGGUAGUAGGUUCUAUAGU	guide forms mismatched pair in position 15 with T-tRNA
21 nt gRNA_mm16	UGAGGUAGUAGGUUGAAUAGU	guide forms mismatched pair in position 16 with T-tRNA
21 nt gRNA_mm17	UGAGGUAGUAGGUUGUUUAGU	guide forms mismatched pair in position 17 with T-tRNA
21 nt gRNA_mm18	UGAGGUAGUAGGUUGUAAAGU	guide forms mismatched pair in position 18 with T-tRNA
21 nt gRNA_mm19	UGAGGUAGUAGGUUGUAUUGU	guide forms mismatched pair in position 19 with T-tRNA
21 nt gRNA_mm20	UGAGGUAGUAGGUUGUAUACU	guide forms mismatched pair in

		position 20 with T-tRNA
21 nt gRNA_mm21	UGAGGUAGUAGGUUGUAUAGA	guide forms mismatched pair in position 21 with T-tRNA
12 nt T-gDNA	TGAGGTAGTAGG	12 nt guide pair with T-tDNA/T-tRNA
13 nt T-gDNA	TGAGGTAGTAGGT	13 nt guide pair with T-tDNA/T-tRNA
14 nt T-gDNA	TGAGGTAGTAGGTT	14 nt guide pair with T-tDNA/T-tRNA
15 nt T-gDNA	TGAGGTAGTAGGTTG	15 nt guide pair with T-tDNA/T-tRNA
16 nt T-gDNA	TGAGGTAGTAGGTTGT	16 nt guide pair with T-tDNA/T-tRNA
17 nt T-gDNA	TGAGGTAGTAGGTTGTA	17 nt guide pair with T-tDNA/T-tRNA
18 nt T-gDNA	TGAGGTAGTAGGTTGTAT	18 nt guide pair with T-tDNA/T-tRNA
19 nt T-gDNA	TGAGGTAGTAGGTTGTATA	19 nt guide pair with T-tDNA/T-tRNA
20 nt T-gDNA	TGAGGTAGTAGGTTGTATAG	20 nt guide pair with T-tDNA/T-tRNA
21 nt T-gDNA	TGAGGTAGTAGGTTGTATAGT	21 nt guide pair with T-tDNA/T-tRNA
25 nt T-gDNA	TGAGGTAGTAGGTTGTATAGTAAGC	25 nt guide pair with T-tDNA/T-tRNA
30 nt T-gDNA	TGAGGTAGTAGGTTGTATAGTAAGCT TGGC	30 nt guide pair with T-tDNA/T-tRNA
40 nt T-gDNA	TGAGGTAGTAGGTTGTATAGTAAGCT TGGCACTGGCCGTC	40 nt guide pair with T-tDNA/T-tRNA

12 nt U-gRNA	UGAGGUAGUAGG	12 nt guide pair with T-tDNA/T-tRNA
13 nt U-gRNA	UGAGGUAGUAGGU	13 nt guide pair with T-tDNA/T-tRNA
14 nt U-gRNA	UGAGGUAGUAGGUU	14 nt guide pair with T-tDNA/T-tRNA
15 nt U-gRNA	UGAGGUAGUAGGUUG	15 nt guide pair with T-tDNA/T-tRNA
16 nt U-gRNA	UGAGGUAGUAGGUUGU	16 nt guide pair with T-tDNA/T-tRNA
17 nt U-gRNA	UGAGGUAGUAGGUUGUA	17 nt guide pair with T-tDNA/T-tRNA
18 nt U-gRNA	UGAGGUAGUAGGUUGUAU	18 nt guide pair with T-tDNA/T-tRNA
19 nt U-gRNA	UGAGGUAGUAGGUUGUAUA	19 nt guide pair with T-tDNA/T-tRNA
20 nt U-gRNA	UGAGGUAGUAGGUUGUAUAG	20 nt guide pair with T-tDNA/T-tRNA
21 nt U-gRNA	UGAGGUAGUAGGUUGUAUAGU	21 nt guide pair with T-tDNA/T-tRNA
25 nt U-gRNA	UGAGGUAGUAGGUUGUAUAGUAAGC	25 nt guide pair with T-tDNA/T-tRNA
30 nt U-gRNA	UGAGGUAGUAGGUUGUAUAGUAAGCUUGGC	30 nt guide pair with T-tDNA/T-tRNA
40 nt U-gRNA	UGAGGUAGUAGGUUGUAUAGUAAGCUUGGCACUGGCCGUC	40 nt guide pair with T-tDNA/T-tRNA
45 GC-F	AAAAGUGCUCAUCAUUGG	18 nt guide pair with Plasmid
45 GC-R	UUCCAAUGAUGAGCACUU	18 nt guide pair with Plasmid
3’-13nt guide DNA	AAGCTTGGCACTG	Guide extending from the 3’ end

		to the 5' end
3'-15nt guide DNA	GTAAGCTTGGCACTG	Guide extending from the 3' end to the 5' end
3'-17nt guide DNA	TAGTAAGCTTGGCACTG	Guide extending from the 3' end to the 5' end
3'-19nt guide DNA	TATAGTAAGCTTGGCACTG	Guide extending from the 3' end to the 5'end
3'-21nt guide DNA	TGTATAGTAAGCTTGGCACTG	Guide extending from the 3' end to the 5' end
3'-25nt guide DNA	AGGTTGTATAGTAAGCTTGGCACTG	Guide extending from the 3'end to the 5' end
3'-13nt guide RNA	AAGCUUGGCACUG	Guide extending from the 3' end to the 5' end
3'-15nt guide RNA	GUAAGCUUGGCACUG	Guide extending from the 3' end to the 5' end
3'-17nt guide RNA	UAGUAAGCUUGGCACUG	Guide extending from the 3'end to the 5' end
3'-19nt guide RNA	UAUAGUAAGCUUGGCACUG	Guide extending from the 3' end to the 5' end
3'-21nt guide RNA	UGUAUAGUAAGCUUGGCACUG	Guide extending from the 3' end to the 5' end
3'-25nt guide RNA	AGGUUGUAUAGUAAGCUUGGCACU G	Guide extending from the 3' end to the 5' end
20 nt Target DNA	TATACAACCTACTACCTCAT	Target DNA for structural prediction
20 nt Target RNA	UAUACAACCUACUACCUCAU	Target RNA for structural prediction
25 nt Target RNA	UAUACAACCUACUACCUCAUACCUC	Target RNA for structural

		prediction
18 nt guide RNA	UGAGGUAGUAGGUUGUAU	guide RNA for structural prediction
18 nt guide DNA	TGAGGTAGTAGGTTGTAT	guide DNA for structural prediction

Supplementary Table S2. List of the ten reads with the highest kurtosis.

Start	End	Gene_id	Gene_name	FPKM	EGGNOG_class_annotation	NR_annotation
335957	337039	gene-B21_RS01580.gene	lacI	33979.94	Transcription	Lactose operon repressor, partial [*Escherichia coli*]
541510	541680	gene-B21_RS02595.gene	ninE	4314.66	--	MULTISPECIES: NinE family protein [*Enterobacteriaceae*]
748730	749812	gene-B21_RS03700.gene	lacI	33979.94	Transcription	Lactose operon repressor, partial [*Escherichia coli*]
1211143	1211316	gene-B21_RS06120.gene	ymgI	2965.56	--	MULTISPECIES: hypothetical protein [*Enterobacteriaceae*]
1578505	1578708	gene-B21_RS07970.gene	ydfZ	1750.5	Function unknown	selenoprotein [*Escherichia coli*]
1596391	1596585	gene-B21_RS08120.gene	xisR	1823.42	Function unknown	excisionase family protein [*Escherichia coli*]
4170243	4170485	gene-B21_RS20640.gene	pspG	1636.73	--	MULTISPECIES: envelope stress response protein PspG [*Enterobacteriaceae*]
537284	537433	gene-B21_RS25125.gene	ylcJ	3671.41	--	MULTISPECIES: hypothetical protein

						[*Enterobacteriaceae*]
1294374	1294547	gene-B21_RS25205.gene	yciY	1617.58	--	hypothetical protein C3441_01795 [*Escherichia coli*]
1345537	1345701	gene-B21_RS25220.gene	B21_RS25220	4501.74	--	MULTISPECIES: protein YmjE [*Bacteria*]

FPKM: Fragments Per Kilobase of exon model per Million mapped fragments. FPKM = {cDNA Fragments \over {Mapped Fragments (Millions) * TranscriptLength (kb)}}.
EGGNOG_class_annotation: Evolutionary Genealogy of Genes: Non-supervised Orthologous Groups class annotation.
NR_annotation: Non-Redundant annotation.

Supplementary Table S3. List of gDNAs and gRNAs targeting HIV-1 ΔDIS 5′UTR RNA.

gDNA #	Sequence (5’-3’)	Target region	5’ product length (nt)
gDNA_1	AGCCAGAGAGCTCCCA	31-46	36
gDNA_2	ACTCAAGGCAAGCTTT	77-92	82
gDNA_3	GACGGGCACACACTAC	100-115	105
gDNA_4	CTAGTTACCAGAGTCA	123-138	128
gDNA_5	GACTAAAAGGGTCTGA	146-161	151
gDNA_6	CACTGCTAGAGATTTT	169-184	174
gDNA_7	CTTTCAAGTCCCTGTT	192-207	197
gDNA_8	GATCTCCTCTGGCTTT	215-230	220
gDNA_9	GCAAGCCGAGTCCTGC	238-253	243
gDNA_10	CCCCTCGCCTCTTGCC	261-276	266
gDNA_11	TTTGGCGTACTCACCA	284-299	289
gDNA_12	TCTAGCCTCCGCTAGT	307-322	312
gRNA_1	AGCCAGAGAGCUCCCAGGCUC	26-46	36
gRNA_2	ACUCAAGGCAAGCUUUAUUGA	72-92	82
gRNA_3	GACGGGCACACACUACUUUGA	95-115	105
gRNA_4	CUAGUUACCAGAGUCACACAA	118-138	128
gRNA_5	GACUAAAAGGGUCUGAGGGAU	141-161	151
gRNA_6	CACUGCUAGAGAUUUUCCACA	164-184	174
gRNA_7	CUUUCAAGUCCCUGUUCGGGC	187-207	197
gRNA_8	GAUCUCCUCUGGCUUUCUUUC	210-230	220
gRNA_9	GCAAGCCGAGUCCUGCUCGAG	233-253	243
gRNA_10	CCCCUCGCCUCUUGCCGUGCG	256-276	266
gRNA_11	UUUGGCGUACUCACCAGUCGC	279-299	289
gRNA_12	UCUAGCCUCCGCUAGUCAAAA	302-322	312
